# Educational interventions to improve literature searching skills in the health sciences: a scoping review

**DOI:** 10.5195/jmla.2020.954

**Published:** 2020-10-01

**Authors:** Julian Hirt, Thomas Nordhausen, Jasmin Meichlinger, Volker Braun, Adelheid Zeller, Gabriele Meyer

**Affiliations:** 1 julian.hirt@fhsg.ch, Institute of Applied Nursing Science, Department of Health, FHS St. Gallen, University of Applied Sciences, St. Gallen, Switzerland, and Medical Faculty, International Graduate Academy, Institute for Health and Nursing Science, Martin Luther University Halle-Wittenberg, Halle (Saale), Germany; 2 thomas.nordhausen@uk-halle.de, Medical Faculty, International Graduate Academy, Institute for Health and Nursing Science, Martin Luther University Halle-Wittenberg, Halle (Saale), Germany; 3 jasmin.meichlinger@fhsg.ch, Institute of Applied Nursing Science, Department of Health, FHS St. Gallen, University of Applied Sciences, St. Gallen, Switzerland; 4 Volker.Braun@medma.uni-heidelberg.de, Medical Faculty, Library of the University Hospital Mannheim, University of Heidelberg, Mannheim, Germany; 5 heidi.zeller@fhsg.ch, Institute of Applied Nursing Science, Department of Health, FHS St. Gallen, University of Applied Sciences, St. Gallen, Switzerland; 6 gabriele.meyer@uk-halle.de, Medical Faculty, International Graduate Academy, Institute for Health and Nursing Science, Martin Luther University Halle-Wittenberg, Halle (Saale), Germany

## Abstract

**Objective:**

The authors reviewed educational interventions for improving literature searching skills in the health sciences.

**Methods:**

We performed a scoping review of experimental and quasi-experimental studies published in English and German, irrespective of publication year. Targeted outcomes were objectively measurable literature searching skills (e.g., quality of search strategy, study retrieval, precision). The search methods consisted of searching databases (CINAHL, Embase, MEDLINE, PsycINFO, Web of Science), tracking citations, free web searching, and contacting experts. Two reviewers performed screening and data extraction. To evaluate the completeness of reporting, the Template for Intervention Description and Replication (TIDieR) was applied.

**Results:**

We included 6 controlled trials and 8 pre-post trials from the 8,484 references that we screened. Study participants were students in various health professions and physicians. The educational formats of the interventions varied. Outcomes clustered into 2 categories: (1) developing search strategies (e.g., identifying search concepts, selecting databases, applying Boolean operators) and (2) database searching skills (e.g., searching PubMed, MEDLINE, or CINAHL). In addition to baseline and post-intervention measurement, 5 studies reported follow-up. Almost all studies adequately described their intervention procedures and delivery but did not provide access to the educational material. The expertise of the intervention facilitators was described in only 3 studies.

**Conclusions:**

The results showed a wide range of study populations, interventions, and outcomes. Studies often lacked information about educational material and facilitators' expertise. Further research should focus on intervention effectiveness using controlled study designs and long-term follow-up. To ensure transparency, replication, and comparability, studies should rigorously describe their intervention.

## INTRODUCTION

Identifying and evaluating available scientific evidence by means of literature review is one component of evidence-based practice (EBP) in health care [1]. Systematic reviews often are the bases of wide-ranging, important decisions in clinical practice [2]. Therefore, literature searches of high methodological quality have considerable scientific, practical, and ethical relevance. Constructing appropriate search strategies to retrieve relevant literature for answering clinical questions is fundamental to systematic reviews. Indeed, effective searching is a core competency of EBP for health sciences professionals, researchers, and librarians [3–5].

In practice, however, many systematic reviews do not employ high-quality literature searches. Several analyses demonstrate shortcomings of literature searches described by published systematic reviews. For example, 7–9 out of 10 search strategies in Cochrane reviews or other systematic reviews contain at least 1 error, such as missing Medical Subject Headings (MeSH), unwarranted explosion of MeSH terms, irrelevant MeSH or free-text terms, missed spelling variants, failure to tailor the search strategy for other databases, and misuse of logical operators [6, 7]. Furthermore, 50%–80% of these errors potentially lower the recall of relevant studies and may impact the overall results of the review [6–8].

Education in EBP should integrate training on literature searching [3]. A recent systematic review indicates that training can improve evidence-based knowledge and skills among health care professionals [9]. However, the actual impact of such training on EBP remains unclear, largely because its implementation and patient-relevant outcomes are rarely examined or are affected by risk of bias [9, 10]. Although training in EBP might improve the skills of health professionals [9], the complexity of these interventions precludes drawing conclusions about their impact on literature searching skills. EBP training can contain several intervention components, such as (1) formulating a research question, (2) developing a search strategy, (3) critically appraising the evidence, and (4) communicating research results to patients. These educational interventions and their components are characterized by varying durations and intensities of training as well as differing materials and methods of delivery [9].

Literature searching skills are, therefore, one outcome of interest among others, and the complexity of EBP training makes it challenging to isolate components and outcomes specifically related to literature searching skills. An older review published in 2003 found some evidence for a positive impact of training on health professionals' skill levels in literature searching; however, the included studies suffered from methodological shortcomings and were underpowered [11]. To scope educational interventions for improving literature searching skills in the health sciences, an updated review is warranted. Therefore, the objective of the current review was to answer the following research questions: What is known about educational interventions to improve literature searching skills in the health sciences? Which outcomes were measured? How completely are these interventions reported in the published literature?

## METHODS

The authors conducted a scoping review following the stages defined by Arksey and O'Malley to (1) map the available evidence relevant to our broad research questions, (2) describe the number and characteristics of available studies, and (3) evaluate the design and reporting of these studies [12–17]. We used an internal review protocol developed by all authors to guide the process and applied PRISMA-ScR for reporting [18].

### Eligibility criteria

We included journal articles that described experimental or quasi-experimental studies in English or German, irrespective of publication year, as we were interested in all available studies focusing on educational interventions with outcome evaluation. The populations of interest were researchers, students, and librarians in the health sciences and health professionals such as nurses, physicians, and pharmacists, regardless of their searching experience or expertise. We included any type of educational intervention (e.g., training, instruction, course, information, peer review) aimed at improving health sciences–related literature searching skills.

We were interested in outcomes that were objectively measurable in terms of improvements in literature searching skills (e.g., proficiency, quality or correctness of search strategies, study retrieval). We excluded studies in which literature searching was one skill or intervention component among others (e.g., EBP courses, courses on reviewing the literature) and those concerning the performance or effectiveness of search filters, hand searching, or citation tracking. We also excluded studies addressing subjective outcomes (e.g., self-perceived knowledge and confidence in literature searching skills), as there is evidence of a weak correlation between self-perceived and objectively assessed literature searching skills [19]. In summary, we were interested in outcomes indicating an impact on literature searching performance.

### Information sources

We searched CINAHL, Embase, MEDLINE via PubMed, Web of Science Core Collection, and PsycINFO via Ovid. Additionally, we performed free web searching via Google Scholar and hand searching in reviews that were identified as relevant. We also contacted expert health sciences librarians through four email lists (Netzwerk Fachbibliotheken Gesundheit, Canadian Medical Libraries, Expertsearch, and MEDBIB-L/German-speaking medical librarians). By forward and backward citation tracking of the included studies, we retrieved additional references. For citation tracking, we used Scopus because this database seemed to cover the largest number of relevant citations for the purpose of this review [20]. Based on already included studies, we searched for more references using the “cited by” and “citing” buttons in Scopus. We exported the retrieved references and, after de-duplication, performed the study selection process as described below. We repeated this process using “cited by” and “citing” buttons in Scopus, if newly identified studies were eligible, until no further studies were included.

### Search

The search strategy was based on database-specific controlled vocabulary and free-text terms. We identified initial search terms on the basis of our experience, an orienting search, and familiar literature. To identify relevant search terms and synonyms, we used the MeSH Browser, COREMINE Medical, and a thesaurus. We analyzed keywords in relevant publications and similar articles that we identified via PubMed to determine frequently occurring terms to include in the search strategy. Considering the presence of delays in keyword indexing, we also searched for controlled vocabulary terms in title and abstract search fields [21]. The search strategy is provided in supplemental Appendix A and Appendix B.

### Selection of sources of evidence

Two authors (Hirt, Nordhausen) designed the search strategy after consultation with the senior authors (Zeller, Meyer). An information specialist (Braun) reviewed the search strategy by means of the Peer Review of Electronic Search Strategy (PRESS) [5]. One author (Hirt) conducted the search. Two independent authors (Hirt, Nordhausen) screened titles, abstracts, and full texts for inclusion using Rayyan and Citavi. We discussed conflicts until reaching consensus.

### Data charting process

Two authors (Hirt, Nordhausen) developed a standardized data extraction sheet. One author (Meichlinger) extracted data on study design, country, setting, participants (i.e., number, gender, age), intervention and control characteristics, outcome measurement, time of measurement, and main results. A second author (Hirt or Nordhausen) checked data extraction. We clarified uncertainties by consulting with the senior authors (Zeller, Meyer). To determine the completeness of reporting in included studies, we applied the Template for Intervention Description and Replication (TIDieR) [22]. For this purpose, two authors (Hirt, Nordhausen) defined a set of minimum required information to meet a TIDieR criterion (supplemental Appendix C). One author (Meichlinger) assessed the reporting of interventions, which was checked by a second author (Hirt or Nordhausen).

### Synthesis of results

One author (Hirt) narratively summarized study characteristics, interventions, outcomes, results, and the reporting of interventions.

## RESULTS

### Search and study selection

**Figure 1:**
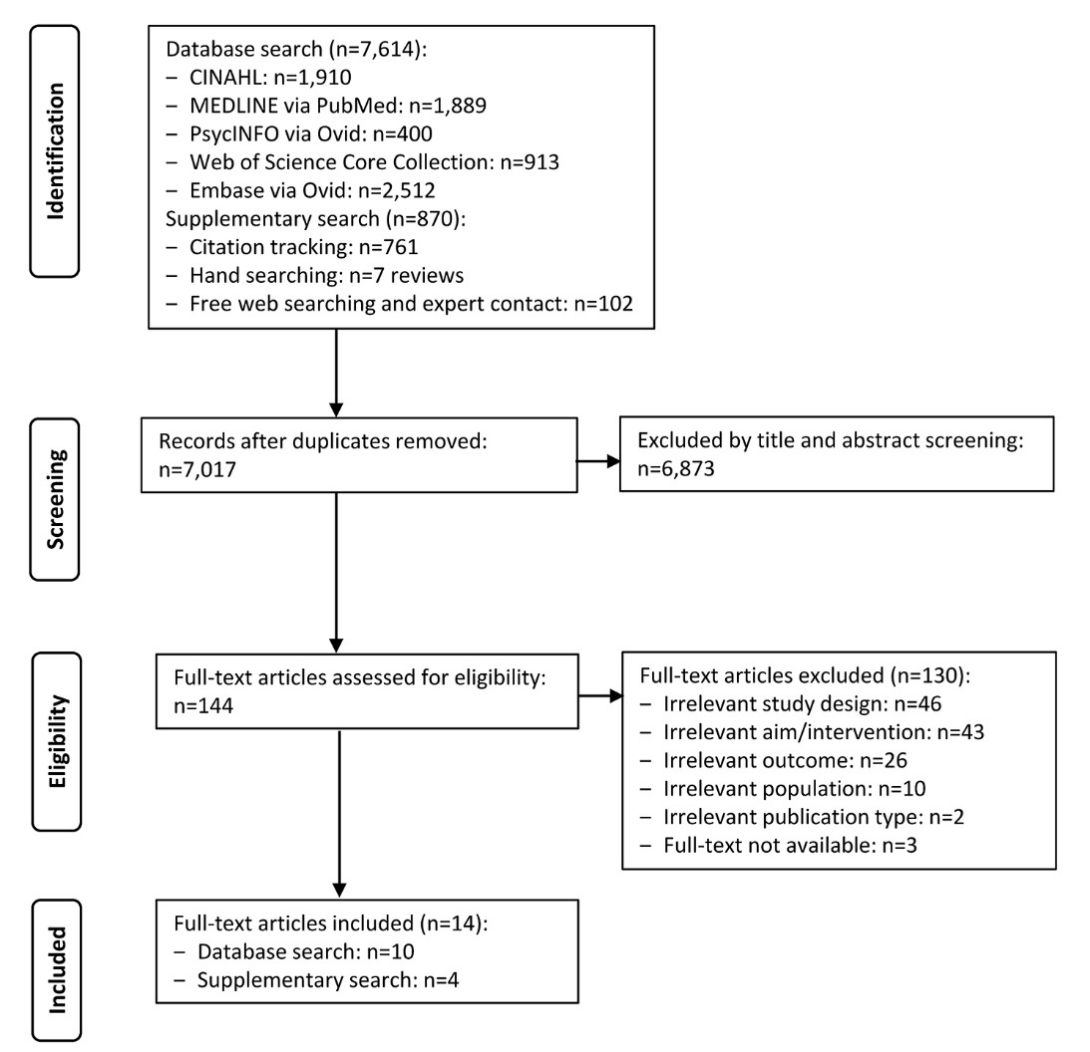
Search and selection process

The systematic database and supplementary search yielded 8,484 references. After removal of duplicates, we screened 7,017 titles and abstracts and 144 full texts, resulting in the inclusion of 14 studies (Figure 1).

### Study characteristics

The included studies (Table 1, with additional details provided in supplemental Appendix D) were published between 1989 and 2019 and were conducted in the United States [23–30], Canada [31, 32], United Kingdom [33, 34], Australia [35], and Pakistan [36]. Eight studies were pre-post trials [24, 27–31, 33, 36], 3 were randomized controlled trials (RCTs) [23, 32, 34], and 3 were controlled trials [25, 26, 35]. One RCT had 2 intervention groups [23]. Most studies were performed in a university setting [24–26, 29–31, 33–36], whereas 4 were conducted in a hospital setting [23, 27, 28, 32]. Participants were physicians or physicians-in-training in 3 studies [23, 28, 32] and students of different health sciences fields in the other studies. The number of participants ranged between 9 and 300 (mean, 77 participants; median, 42 participants). Most studies did not report participants' gender or age.

**Table 1:** Characteristics, interventions, and outcomes of included studies

Reference	Design	Participants	(n)	Intervention	Outcome
Erickson and Warner, 1998 [23]	RCT	Residents in obstetrics and gynecology	31	IG1: Hands-on tutorial session on the use of MEDLINE by health sciences librarian with hands-on instruction IG2: Tutorial on the use of MEDLINE at prescribed sessions performed by health sciences librarian	MEDLINE search recall and precision rates of 4 searches
Hobbs et al., 2015 [24]	Pre-post trial	Senior undergraduate radiologic science students	17	Library instruction on planning literature searches, developing search strategies, searching health-related and medicine-related databases	Questionnaire on knowledge and skills in information literacy developed by the health sciences librarian and the Radiologic Science Faculty
Carlock and Anderson, 2007 [25]	CT	Undergraduate nursing students	90	Librarian instruction including homework and in-class assignment	Self-created rubric comparing the search history of a predefined search in CINAHL
Gruppen, et al., 2005 [26]	CT	Fourth-year medical students	92	Instructional intervention on EBP-based techniques for searching MEDLINE for evidence related to a clinical problem taught by medical librarians	Structured clinical scenario (described in a publication) and scoring on literature search quality and search errors developed by librarians at the University of Michigan, based on a template designed by librarians at the University of Rochester
Rosenfeld et al., 2002 [27]	Pre-post trial	Intensive care unit nurses	36	Educational sessions complemented by a web-tutorial regarding information literacy competencies, performed by the medical librarian	Self-defined, point-based competency rating scale
Vogel et al., 2002 [28]	Pre-post trial	Second-year medicine residents	42	Workshop on using Ovid's version of MEDLINE	Participants completed the MEDLINE performance checklist
Grant et al., 1996 [29]	Pre-post trial	Pharmacy students	48	Lecture on systematic approach in combination with an online demonstration with OVID to develop search strategies and homework assignments to perform a literature search	Evaluation of 2 written search strategies (one sensitive, one specific) concerning a predefined research question by pre-established scoring criteria
Bradigan and Mularski, 1989 [30]	Pre-post trial	Second-year medical students	9	Mini module courses performed by 2 librarian instructors	Number of correct answers, 3 questions on the ability to extract important concepts in a statement of a medical problem to be searched online
Sikora et al., 2019 [31]	Pre-post trial	Undergraduate or graduate health sciences or medical students	29	Scheduled individualized research consultations for students, performed by librarians	Self-developed information literacy rubric for scoring of open-ended questions regarding the use of appropriate keywords and search strategies
Haynes et al., 1993 [32]	RCT	Physicians and physicians-in-training	264	Feedback on the first 10 searches and assignment by a clinical MEDLINE preceptor	Participants performed 10 MEDLINE searches concerning individual research questions; the percentage of successful searches was defined if at least 1 relevant reference was retrieved
Grant and Brettle, 2006 [33]	Pre-post trial	Postgraduate students in research in health and social care	13	Self-developed web-based MEDLINE tutorial by an information specialist and tutor	Modified Rosenberg assessment tool comprised a skills checklist
Brettle and Raynor, 2013 [34]	RCT	Undergraduate nursing students	55	Online in-house information literacy tutorial (session 1) and follow-up information skills session (face-to-face) after one month (session 2)	Test of skills to search for evidence via CINAHL concerning specific research questions using a rubric identifying key features in the search strategy
Wallace et al., 2000 [35]	CT	Undergraduate nursing, health, and behavioral sciences students	300	Curriculum-integrated information literacy program	Objective test of library catalog skills regarding 5 domains
Qureshi et al., 2015 [36]	Pre-post trial	Postgraduate dental students	42	Workshop comprising 3 sessions of lectures and hands-on practice	Questions of the Fresno Test tool

Abbreviations: CT=Controlled trial; EBP=Evidence-based practice; IG=Intervention group; MeSH=Medical Subject Headings; NA=Not applicable; RCT=Randomized controlled trial.

### Intervention characteristics

Intervention groups received an instructional session [23, 24, 26], an instructional session combined with homework [25], an instructional or educational session complemented by a web-based tutorial [27], a consultation [31], training and feedback [32], a workshop [28, 36], a course [30], a curriculum-integrated program [35], a lecture [33], an online tutorial with a face-to-face follow-up session [34], a hands-on tutorial [23], or a web-based tutorial [33]. Control groups received a face-to-face session and follow-up sessions [34], training without feedback [33], or no intervention [23, 26, 32, 35].

### Study outcomes

Outcomes clustered into two groups: (1) search strategy development and (2) database searching skills (Table 2). Beside baseline and post-intervention measurement, five studies reported follow-up outcomes one to eleven months later [26, 28, 29, 32, 33].

**Table 2:** Outcomes clustered in two categories: (1) search strategy development and (2) database search skills

Search strategy development	Database searching skills
Using appropriate search strategies	Showing database search skills in general
Using appropriate keywords	Searching more than one database
Developing search strategies in general	Creating sensitive and specific search strategies using Ovid
Identifying search concepts	Retrieving a manageable number of references in MEDLINE
Selecting databases	Having recall and precision in MEDLINE
Applying Boolean operators	Searching PubMed
Applying indexing terms	Searching MEDLINE
Applying search limits	Searching CINAHL
Search quality and errors	Searching a library catalog

Outcomes related to developing search strategies included using appropriate keywords and appropriate search strategies [31]; developing search strategies in general [24]; identifying search concepts [24, 30]; selecting databases and where to find evidence [24, 34, 36]; applying Boolean operators, indexing terms, and search limits [27, 30, 33]; and having search quality as well as errors [26].

Outcomes related to database searching skills included database searching skills in general or searching more than one database [24, 27]; creating sensitive and specific search strategies using Ovid [29]; retrieving a manageable and relevant number of references in MEDLINE [33]; having recall and precision in MEDLINE [23]; searching PubMed [36], MEDLINE [28, 32], CINAHL [25, 34]; and searching a library catalog [35].

### Study results

Most of the included studies—which consisted of pre-post trials, RCTs, and controlled trials—reported positive outcomes (i.e., an improvement in literature searching skills). Some pre-post trials reported significant improvements in the development of search strategies [31, 33, 36] and database searching skills [33, 36]. Other pre-post trials reported non-significant improvements in the development of search strategies [24, 27, 30] and database searching skills [24, 27, 29]. One study reported a significant improvement without reporting descriptive data concerning database searching skills [28].

Concerning RCTs and controlled trials, one reported a significant improvement in database searching skills [34], whereas others reported non-significant improvements in database searching skills [25, 32, 35]. Two studies reported no improvements in database searching skills [23, 34]. In one study, the intervention group had a significantly higher search quality score and significantly lower number of search errors [26].

### Reporting of interventions

In six studies, the educational materials that were used were not clearly described [24–26, 34–36]. Almost all studies adequately reported the procedures of the interventions, except for two [31, 32]. Only three studies characterized the persons who delivered the intervention in terms of their professional background, expertise, and/or specific training [23, 31, 32]. All studies reported how the intervention was delivered. Four studies did not report the location of the intervention [23, 29, 31, 34]. Three studies did not report the number or frequency of educational sessions [26, 31, 35]. Most studies did not report tailoring, except for three studies [26, 31, 33], or modifications of the intervention or its planned and actual intervention fidelity, except for one [34] (Table 3).

**Table 3:** Reporting assessment of included studies with Template for Intervention Description and Replication (TIDieR) (n=14)

	What: materials	What: procedures	Who provided	How	Where	When and how often	Tailoring	Modifications	How well (planned)	How well (actual)
Erickson and Warner, 1998 [23]	Y	Y	Y	Y	N	Y	—	—	—	—
Hobbs et al., 2015 [24]	N	Y	N	Y	Y	Y	—	—	—	—
Carlock and Anderson, 2007 [25]	N	Y	N	Y	Y	Y	—	—	—	—
Gruppen, et al., 2005 [26]	N	Y	N	Y	Y	N	Y	—	—	—
Rosenfeld et al., 2002 [27]	Y	Y	N	Y	Y	Y	—	—	—	—
Vogel et al., 2002 [28]	Y	Y	N	Y	Y	Y	—	—	—	—
Grant et al., 1996 [29]	Y	Y	N	Y	N	Y	—	—	—	—
Bradigan and Mularski [30]	Y	Y	N	Y	Y	Y	—	—	—	—
Sikora et al., 2019 [31]	Y	N	Y	Y	N	N	Y	—	—	—
Haynes et al., 1993 [32]	Y	N	Y	Y	Y	Y	—	—	—	—
Grant and Brettle, 2006 [33]	Y	Y	N	Y	Y	Y	Y	—	—	—
Brettle and Raynor, 2013 [34]	N	Y	N	Y	N	Y	—	—	—	Y
Wallace et al., 2000 [35]	N	Y	N	Y	Y	N	—	—	—	—
Qureshi et al., 2015 [36]	N	Y	N	Y	Y	Y	—	—	—	—

Abbreviations: Y=Yes, reported; N=No, not reported; —=Unclear whether it was conducted.

## DISCUSSION

This scoping review focused on intervention studies aiming to improve literature searching skills in the health sciences. The results showed a wide range of study populations, educational interventions and their components, and outcomes. Overall, the reporting of these studies lacked essential details about educational materials and intervention deliveries to allow a detailed understanding of how the educational interventions were applied.

Database searching is a key element of systematic literature searches [37]. Systematic reviews require a high methodological standard of literature searching using several databases and highly sensitive search strategies [38]. Eight of the fourteen included studies tested database-specific educational interventions to improve searching skills in CINAHL, MEDLINE, and PubMed [23, 25, 28, 29, 32–34, 36]. Since the advent of electronic databases in the late 1970s, technological progress and usability requirements resulted in fundamental changes in database functionalities, capabilities, and layouts [39]. These changes were paralleled by changes in search features and user interfaces. Recent examples are the launch of “new PubMed” as well as updated search functions and layout of the Cochrane Library. These evolutions should be taken into account when designing contemporary educational interventions to improve literature searching skills. Only two studies with database-specific educational interventions were published within the last ten years [34, 36]; thus, the timeliness of these interventions is questionable.

Furthermore, there was general lack of complete reporting among the included studies, including detailed descriptions of database-specific educational interventions and outcomes. While the intervention procedure, format, and intensity (i.e., when and how much) were often reported, most studies lacked information about the educational material used and the expertise of the persons delivering the intervention. These two aspects—timeliness and transparent reporting of interventions—might hinder the transfer of educational interventions into current practice [40]. To better describe the context of implementation of educational interventions and ensure study transparency, replicability, adaptation, and comparability, authors should thoroughly describe their interventions [9] using TIDieR [22] or CReDECI-2 [41] guidelines.

Participants in the included studies consisted of students or physicians. Thus, these studies mainly addressed university education and clinical practice. One finding of our review was that experimental and quasi-experimental studies on objectively measurable literature searching skills of librarians as study participants were lacking, despite the increasing demand to involve skilled librarians and information specialists as methodological peer reviewers or as part of systematic review teams [42, 43]. However, there is evidence that underlines the importance of the active involvement of expert librarians in systematic reviews. A recently published analysis showed that systematic reviews coauthored by librarians had less risk of bias than reviews in which librarians' contributions were only mentioned in the acknowledgments or were unclear [44]. A recently developed competency framework for librarians who are involved in systematic reviews contains indicators that are helpful for determining whether the tasks performed by librarians could be applied independently [45].

Furthermore, a discussion-based framework presenting propositions for planning, developing, and evaluating training interventions for expert librarians to participate effectively in systematic review teams was recently published [46]. These two contextual propositions might underpin the theoretical framework for effective involvement of expert librarians in systematic review teams. However, based on the results of our scoping review, it remains unclear how librarians' searching skills might be further developed or improved. Given their increasingly important role in systematic reviewing, the effectiveness of literature search skill training for librarians should be considered in future research [46].

The development of feasible and effective interventions should be accompanied by the selection of relevant outcomes that are measured at appropriate times [47]. The included studies involved different outcomes, but their times of measurement were predominantly baseline and post-intervention. Follow-up measurement was conducted only in five studies, with a wide range between one and eleven months. Therefore, it is difficult to draw conclusions regarding long-term effects of educational interventions. Studies indicate that literature searching skills can worsen over time [48]. Future research should, therefore, employ long-term evaluations to ensure that the outcomes of interventions demonstrate continuity and long-term effectiveness. This might also promote the development of enduring information material such as manuals that guide students, researchers, and librarians in systematic literature searching.

Literature search recall and precision can be improved by other potential interventions including the development of search filters, supplementary search methods such as hand searching and citation tracking, structured search forms, and objectively derived search terms [37, 49–52]. These interventions were excluded due to our focus on educational interventions. However, such interventions need to be considered in systematic literature searching effectiveness [53].

The included studies were not critically appraised [18]. Nevertheless, significant limitations of internal and external validity were evident. Most studies were neither controlled nor randomized and had small sample sizes. The presentation of results was insufficient in some studies (e.g., confidence intervals and/or estimated effect sizes were not reported). Furthermore, the included studies used different types of outcomes, impeding comparison of their results. To enhance further research, authors should ensure objective, valid, and reliable outcome measurement [54], which could lead to greater internal validity and comparability of results.

A strength of our scoping review was our comprehensive search using a peer-reviewed search strategy [5]. To transform our research question into a search strategy, we intended to use two search components: education and literature search. However, this resulted in a large number of search results that we could not feasibly work through considering our resources. To further limit the search results, we added three concepts under the guidance of a medical expert librarian (Braun): (1) aim of the intervention (e.g., competence, improvement, success); (2) health sciences–related participants (e.g., clinician, librarian, nurse, physician); and (3) study design (e.g., randomized, trial, quasi-experimental). Adding these search components yielded a lower number of results and increased the risk of excluding relevant studies [55]. Therefore, to avoid missing studies that might contribute to the body of evidence, we applied several supplementary search methods—including citation tracking, free web searching, hand searching in relevant reviews, and contacting experts [37, 56]—resulting in the identification of four additional studies.

However, a limitation of our search approach was that we did not search trial registries or conference proceedings as well as specialized educational and library-related databases such as Education Resources Information Center (ERIC); Library, Information Science & Technology Abstracts (LISTA); and Library and Information Science Abstracts (LISA). Instead, we focused on databases covering multiple disciplines such as CINAHL, PsycINFO, and Web of Science Core Collection. The non-use of specialized library-related databases was due to limited subscriptions at our institution but may be a reason why no studies describing educational interventions for librarians were identified.

Another strength of our review was that we ensured high data quality by using two independent reviewers for study selection and a second reviewer to check data extraction and assessment of reporting. The analysis using TIDieR enabled a comprehensive assessment of the reporting of educational interventions to improve literature searching skills in health sciences [57]. To increase the reliability of the data extraction, two reviewers (Hirt, Nordhausen) developed minimal requirements for each TIDieR item criterion.

Our research question was limited to experimental and quasi-experimental studies employing search-specific educational interventions to improve literature searching skills. This scoping review did not address the effectiveness of interventions and quality of studies, which was appropriate for scoping review methodology [12, 17]. Conclusions concerning the positive impact of the identified educational interventions are, therefore, limited. The small number of rigorous study designs that we identified has led us to conclude that a systematic review to determine the effectiveness of interventions and internal validity of studies might not be necessary at the moment, as the last RCT was published in 2013 [34]. Further primary research is needed and should focus on the long-term effectiveness of educational interventions to improve literature searching skills using controlled study designs and follow-up outcome measurement.

To understand which interventions are effective for whom and why, the development of educational interventions should follow a systematic process based on available evidence and the needs of end users and librarians. This review indicates that there is still a need for high-quality research employing well-developed educational interventions and contemporary search methods using rigorous study designs [11].

## SUPPLEMENTAL FILES

**Appendix A:** Search components and search terms

**Appendix B:** Database-specific search strategies

**Appendix C:** Characteristics, interventions, outcomes, and main results of included studies

**Appendix D:** Detailed reporting assessment using the Template for Intervention Description and Replication (TIDieR)
