# Supplementary material for: Educational interventions to improve literature searching skills in the health sciences: a scoping review
Source: J Med Libr Assoc. 2020 Oct 1;108(4):534–46. doi: 10.5195/jmla.2020.954 (PMC7524628; doi:10.5195/jmla.2020.954)
Supplement: Supplementary file 1 — Appendix A: Search components and search terms [file jmla-108-4-534-s01.pdf]

JMLA Journal of the Medical Library Association

SC
Supplemental content to
J Med Libr Assoc. Oct;108(4):dx.doi.org/10.5195/jmla.2020.954
www.jmla.mlanet.org
© Hirt, Nordhausen, Meichlinger, Braun, Zeller, Meyer 2020

Educational interventions to improve literature searching skills in the health sciences: a scoping review

Julian Hirt; Thomas Nordhausen; Jasmin Meichlinger; Volker Braun; Adelheid Zeller; Gabriele Meyer

APPENDIX A
Search components and search terms

Component	Term
Intervention	Education*
	Teaching*†
	Program
	Programme
	Material
	Teaching Materials*
	Flyer
	Support
	Help
	Manuals as Topic*
	Manual
	Course
	Curriculum*
	Training
	Coaching
	Mentoring*
	Mentorship
	Guide
	Guidance
	Guideline
	Study Guide as Topic*
	Instruction
	Information
	Consulting
	Consultation
	Schooling
	Learning
	Exercise
	Tutor

SC
Supplemental content to
J Med Libr Assoc. Oct;108(4):dx.doi.org/10.5195/jmla.2020.954
www.jmla.mlanet.org
© Hirt, Nordhausen, Meichlinger, Braun, Zeller, Meyer 2020

Component	Term
	Scholarship<br>Library Services*<br>Peer Review*<br>Peer Group*
Aim of Intervention	Promotion<br>Improvement<br>Quality Improvement*<br>Increase<br>Enhancement<br>Development<br>Evolution<br>Evaluation<br>Upgrade<br>Success<br>Competence<br>Skills<br>Expertise<br>Competency<br>Ability<br>Educational Measurement*<br>Advance<br>Advancement<br>Benefit<br>Gain<br>Progress<br>Achievement<br>Boost
Literature search	Databases as Topic*<br>Databases, Bibliographic*†<br>Bibliography as Topic*<br>Database<br>Database search<br>Database searching<br>Information Literacy*<br>Search Engine*<br>Literature search

Supplemental content to
J Med Libr Assoc. Oct;108(4):dx.doi.org/10.5195/jmla.2020.954
www.jmla.mlanet.org
© Hirt, Nordhausen, Meichlinger, Braun, Zeller, Meyer 2020

Component	Term
	Literature searching
	Information Services*†
	Search result
	Search strategy
	Search string
	Search process
	Information search
	Information searching
	Information seeking
	Information Seeking Behavior*
	Information finding
	Literacy skills
Health sciences	Health Personnel*
	Libraries*
	Libraries, Dental*†
	Libraries, Digital*†
	Libraries, Hospital*†
	Libraries, Medical*†
	National Library of Medicine (U.S.)*†
	Libraries, Nursing*†
	Libraries, Special*†
	Health Occupation*
	Students*
	Allied Health Personnel*†
	Health
	Health professionals
	Library
	Librarian
	Information specialist
	Nurses*†
	Nursing Staff*†
	Nursing*
	Nursing Research*
	Medicine
	Medical
	Physicians*†

Supplemental content to
J Med Libr Assoc. Oct;108(4):dx.doi.org/10.5195/jmla.2020.954
www.jmla.mlanet.org
© Hirt, Nordhausen, Meichlinger, Braun, Zeller, Meyer 2020

Component	Term
	Doctor
	Clinician
	Graduate
	Undergraduate
	Physical Therapists*†
	Physical therapy
	Physiotherapy
	Physiotherapist
	Midwifery*
	Midwife
	Therapy
	Therapist
	Occupational Therapists*†
	Speech Therapy*
	Paramedic
	Psychology*
	Psychologist
	Psychotherapy*
	Psychotherapist
	Pharmacy*
	Pharmacist*†
	Health Science
	Health Scientist
	Health Researcher
Design	Controlled Clinical Trials as Topic*
	Controlled Clinical Trial*
	Non-Randomized Controlled Trials as Topic*†
	Randomized Controlled Trials as Topic*†
	Randomized Controlled Trial*
	Pragmatic Clinical Trials as Topic*†
	Controlled Clinical Trial*
	Randomized Controlled Trial*†
	Pragmatic Clinical Trial*†
	Cross-Over Studies*
	Trial
	Study

JMLA Journal of the Medical Library Association

SC
Supplemental content to
J Med Libr Assoc. Oct;108(4):dx.doi.org/10.5195/jmla.2020.954
www.jmla.mlanet.org
© Hirt, Nordhausen, Meichlinger, Braun, Zeller, Meyer 2020

Component	Term
	Examination
	Exploratory
	Inquiry
	Design
	Approach
	Investigation
	Randomisation
	Randomised
	Randomization
	Randomized
	Cluster-randomisation
	Cluster-randomization
	Cluster-randomised
	Cluster-randomized
	Stepped wedge
	Stepped-wedge
	Quasi-experimental
	Experiment
	Pre-post
	Crossover

This table lists the controlled vocabulary and further search terms as an example for the database MEDLINE, other words than nouns will be integrated by means of truncation and/or other type of words.
* Used as controlled vocabulary.
† Narrower term of already listed one.
