# Supplementary material for: Educational interventions to improve literature searching skills in the health sciences: a scoping review
Source: J Med Libr Assoc. 2020 Oct 1;108(4):534–46. doi: 10.5195/jmla.2020.954 (PMC7524628; doi:10.5195/jmla.2020.954)
Supplement: Supplementary file 2 — Appendix B: Database-specific search strategies [file jmla-108-4-534-s02.pdf]

JMLA Journal of the Medical Library Association

SC

Supplemental content to
J Med Libr Assoc. Oct;108(4):dx.doi.org/10.5195/jmla.2020.954
www.jmla.mlanet.org
© Hirt, Nordhausen, Meichlinger, Braun, Zeller, Meyer 2020

Educational interventions to improve literature searching skills in the health sciences: a scoping review

Julian Hirt; Thomas Nordhausen; Jasmin Meichlinger; Volker Braun; Adelheid Zeller; Gabriele Meyer

APPENDIX B

Database-specific search strategies

CINAHL

Date: January 11, 2019

#	Enter	Hits
1	(MH PEER REVIEW+ OR MH MENTORSHIP+ OR MH EDUCATION+ OR MH INFORMATION RESOURCES+ OR MH LEARNING+ OR TI "PEER REVIEW" OR TI "PEER REVIEWS" OR TI "PEER REVIEWING" OR TI "PEER GROUPS" OR TI MENTORING OR TI EDUCATION OR TI EDUCATIONAL OR TI EDUCATE OR TI EDUCATING OR TI TEACH OR TI TEACHING OR TI PROGRAM OR TI PROGRAMS OR TI PROGRAMME OR TI PROGRAMMES OR TI MATERIAL OR TI MATERIALS OR TI FLYER OR TI SUPPORT OR TI SUPPORTING OR TI HELP OR TI HELPING OR TI MANUAL OR TI MANUALS OR TI COURSE OR TI COURSES OR TI TRAINING OR TI TRAININGS OR TI TRAIN OR TI COACHING OR TI COACHINGS OR TI COACH OR TI MENTORSHIP OR TI MENTORSHIPS OR TI GUIDE OR TI GUIDES OR TI GUIDANCE OR TI GUIDANCES OR TI GUIDELINE OR TI GUIDELINES OR TI INSTRUCTION OR TI INSTRUCTIONS OR TI INSTRUCT OR TI INFORMATION OR TI INFORM OR TI CONSULTING OR TI CONSULTATION OR TI CONSULT OR TI SCHOOLING OR TI LEARNING OR TI LEARN OR TI EXERCISE OR TI TUTOR OR TI SCHOLARSHIP)	1,412,021
2	(MH DATABASES+ OR MH LITERATURE SEARCHING+ OR MH COMPUTERIZED LITERATURE SEARCHING+ OR MH INFORMATION LITERACY+ OR MH INFORMATION RETRIEVAL+ OR MH INFORMATION SEEKING BEHAVIOR+ OR TI "DATABASE SEARCH" OR AB "DATABASE SEARCH" OR TI "DATABASE SEARCHES" OR AB "DATABASE SEARCHES" OR TI "DATABASE SEARCHING" OR AB "DATABASE SEARCHING" OR TI "LITERATURE SEARCH" OR AB "LITERATURE SEARCH" OR TI "LITERATURE SEARCHES" OR AB "LITERATURE SEARCHES" OR TI "LITERATURE SEARCHING" OR AB "LITERATURE SEARCHING" OR TI "SEARCH RESULT" OR AB "SEARCH RESULT" OR TI "SEARCH RESULTS" OR AB "SEARCH RESULTS" OR TI "SEARCH STRATEGY" OR AB "SEARCH STRATEGY" OR TI "SEARCH STRATEGIES" OR AB "SEARCH STRATEGIES" OR TI "SEARCH STRING" OR AB "SEARCH STRING" OR TI "SEARCH STRINGS" OR AB "SEARCH STRINGS" OR TI "SEARCH PROCESS" OR AB "SEARCH PROCESS" OR TI "SEARCH PROCESSES" OR AB "SEARCH PROCESSES" OR TI "INFORMATION SEARCH" OR AB "INFORMATION SEARCH" OR TI "INFORMATION SEARCHES" OR AB "INFORMATION SEARCHES" OR TI "INFORMATION SEARCHING" OR AB "INFORMATION SEARCHING" OR TI "INFORMATION SEEKING" OR AB "INFORMATION SEEKING" OR TI "INFORMATION FINDING" OR AB	129,258

Journal of the Medical Library Association

Supplemental content to
J Med Libr Assoc. Oct;108(4):dx.doi.org/10.5195/jmla.2020.954
www.jmla.mlanet.org
© Hirt, Nordhausen, Meichlinger, Braun, Zeller, Meyer 2020

	"INFORMATION FINDING" OR TI "LITERACY SKILLS" OR AB "LITERACY SKILLS")	
3	(MH QUALITY IMPROVEMENT+ OR TI PROMOTION OR TI PROMOTE OR TI IMPROVEMENT OR TI IMPROVE OR TI INCREASE OR TI ENHANCEMENT OR TI ENHANCE OR TI DEVELOPMENT OR TI DEVELOP OR TI UPGRADE OR TI SUCCESS OR TI ADVANCE OR TI ADVANCEMENT OR TI BENEFIT OR TI GAIN OR TI PROGRESS OR TI ACHIEVEMENT OR TI ACHIEVE OR TI BOOST)	259,753
4	(MH CROSSOVER DESIGN+ OR MH EXPERIMENTAL STUDIES+ OR MH QUASI-EXPERIMENTAL STUDIES+ OR TI TRIAL OR AB TRIAL OR TI TRIALS OR AB TRIALS OR TI STUDY OR AB STUDY OR TI STUDIES OR AB STUDIES OR TI EXAMINATION OR AB EXAMINATION OR TI EXAMINATIONS OR AB EXAMINATIONS OR TI EXAMINATE OR AB EXAMINATE OR TI EXPLORATORY OR AB EXPLORATORY OR TI INQUIRY OR AB INQUIRY OR TI INQUIRIES OR AB INQUIRIES OR TI INVESTIGATION OR AB INVESTIGATION OR TI INVESTIGATIONS OR AB INVESTIGATIONS OR TI INVESTIGATE OR AB INVESTIGATE OR TI APPROACH OR AB APPROACH OR TI DESIGN OR AB DESIGN OR TI RANDOM* OR AB RANDOM* OR TI CLUSTER-RANDOM* OR AB CLUSTER-RANDOM* OR TI "STEPPED WEDGE" OR AB "STEPPED WEDGE" OR TI STEPPED-WEDGE OR AB STEPPED-WEDGE OR TI QUASI-EXPERIMENTAL OR AB QUASI-EXPERIMENTAL OR TI EXPERIMENT OR AB EXPERIMENT OR TI EXPERIMENTS OR AB EXPERIMENTS OR TI EXPERIMENTAL OR AB EXPERIMENTAL OR TI PRE-POST OR AB PRE-POST OR TI "PRE POST" OR AB "PRE POST" OR TI CROSSOVER OR AB CROSSOVER)	2,057,899
5	(MH HEALTH PERSONNEL+ OR MH HEALTH OCCUPATIONS+ OR MH SCIENTISTS+ OR MH RESEARCH PERSONNEL+ OR MH LIBRARIANS+ OR TI "HEALTH PERSONNEL" OR AB "HEALTH PERSONNEL" OR TI "HEALTH PROFESSIONAL" OR AB "HEALTH PROFESSIONAL" OR TI "HEALTH PROFESSIONALS" OR AB "HEALTH PROFESSIONALS" OR TI NURS* OR AB NURS* OR TI LIBRAR* OR AB LIBRAR* OR TI STUDENT OR AB STUDENT OR TI STUDENTS OR AB STUDENTS OR TI MIDWIFE* OR AB MIDWIFE* OR TI PSYCHOLOG* OR AB PSYCHOLOG* OR TI PSYCHOTHERAP* OR AB PSYCHOTHERAP* OR TI PHARMACY OR AB PHARMACY OR TI PHARMACEUTIC* OR AB PHARMACEUTIC* OR TI "INFORMATION SPECIALIST" OR AB "INFORMATION SPECIALIST" OR TI "INFORMATION SPECIALISTS" OR AB "INFORMATION SPECIALISTS" OR TI MEDICINE OR AB MEDICINE OR TI MEDICAL OR AB MEDICAL OR TI DOCTOR OR AB DOCTOR OR TI DOCTORS OR AB DOCTORS OR TI CLINIC OR AB CLINIC OR TI CLINICS OR AB CLINICS OR TI CLINICAL OR AB CLINICAL OR TI CLINICIAN OR AB CLINICIAN OR TI CLINICIANS OR AB CLINICIANS OR TI GRADUATE OR AB GRADUATE OR TI GRADUATES OR AB GRADUATES OR TI UNDERGRADUATE OR AB UNDERGRADUATE OR TI UNDERGRADUATES OR AB UNDERGRADUATES OR TI "PHYSICAL THERAPY" OR AB "PHYSICAL THERAPY" OR TI "PHYSICAL THERAPIES" OR AB "PHYSICAL THERAPIES" OR TI PHYSIOTHERAP* OR AB PHYSIOTHERAP* OR TI THERAP* OR AB THERAP* OR TI PARAMEDIC* OR AB PARAMEDIC* OR TI PSYCHOLOGIST OR AB PSYCHOLOGIST OR TI PSYCHOLOGISTS OR AB PSYCHOLOGISTS OR TI PSYCHOTHERAPIST OR AB PSYCHOTHERAPIST OR TI PSYCHOTHERAPISTS OR AB PSYCHOTHERAPISTS OR TI "HEALTH SCIENCE" OR AB "HEALTH	2,516,874

SC
Supplemental content to
J Med Libr Assoc. Oct;108(4):dx.doi.org/10.5195/jmla.2020.954
www.jmla.mlanet.org
© Hirt, Nordhausen, Meichlinger, Braun, Zeller, Meyer 2020

	SCIENCE" OR TI "HEALTH SCIENCES" OR AB "HEALTH SCIENCES" OR TI "HEALTH SCIENTIST" OR AB "HEALTH SCIENTIST" OR TI "HEALTH SCIENTISTS" OR AB "HEALTH SCIENTISTS" OR TI "HEALTH RESEARCHER" OR AB "HEALTH RESEARCHER" OR TI "HEALTH RESEARCHERS" OR AB "HEALTH RESEARCHERS")	
6	#1 AND #2 AND #3 AND #4 AND #5	1,910

JMLA Journal of the Medical Library Association

Supplemental content to
J Med Libr Assoc. Oct;108(4):dx.doi.org/10.5195/jmla.2020.954
www.jmla.mlanet.org
© Hirt, Nordhausen, Meichlinger, Braun, Zeller, Meyer 2020

MEDLINE via PubMed

Date: January 11, 2019

#	Enter	Hits
1	((EDUCATION[MESH] OR "TEACHING MATERIALS"[MESH] OR "MANUALS AS TOPIC"[MESH] OR "STUDY GUIDE AS TOPIC"[MESH] OR CURRICULUM[MESH] OR MENTORING[MESH] OR "LIBRARY SERVICES"[MESH] OR "PEER REVIEW"[MESH] OR "PEER GROUP"[MESH] OR "PEER REVIEW"[TITLE] OR "PEER REVIEWS"[TITLE] OR "PEER REVIEWING"[TITLE] OR "PEER GROUPS"[TITLE] OR MENTORING[TITLE] OR EDUCATION[TITLE] OR EDUCATIONAL[TITLE] OR EDUCATE[TITLE] OR EDUCATING[TITLE] OR TEACH[TITLE] OR TEACHING[TITLE] OR PROGRAM[TITLE] OR PROGRAMS[TITLE] OR PROGRAMME[TITLE] OR PROGRAMMES[TITLE] OR MATERIAL[TITLE] OR MATERIALS[TITLE] OR FLYER[TITLE] OR SUPPORT[TITLE] OR SUPPORTING[TITLE] OR HELP[TITLE] OR HELPING[TITLE] OR MANUAL[TITLE] OR MANUALS[TITLE] OR COURSE[TITLE] OR COURSES[TITLE] OR TRAINING[TITLE] OR TRAININGS[TITLE] OR TRAIN[TITLE] OR COACHING[TITLE] OR COACHINGS[TITLE] OR COACH[TITLE] OR MENTORSHIP[TITLE] OR MENTORSHIPS[TITLE] OR GUIDE[TITLE] OR GUIDES[TITLE] OR GUIDANCE[TITLE] OR GUIDANCES[TITLE] OR GUIDELINE[TITLE] OR GUIDELINES[TITLE] OR INSTRUCTION[TITLE] OR INSTRUCTIONS[TITLE] OR INSTRUCT[TITLE] OR INFORMATION[TITLE] OR INFORM[TITLE] OR CONSULTING[TITLE] OR CONSULTATION[TITLE] OR CONSULT[TITLE] OR SCHOOLING[TITLE] OR LEARNING[TITLE] OR LEARN[TITLE] OR EXERCISE[TITLE] OR TUTOR[TITLE] OR SCHOLARSHIP[TITLE]))	1,712,625
2	(("DATABASES AS TOPIC"[MESH] OR "INFORMATION LITERACY"[MESH] OR "SEARCH ENGINE"[MESH] OR "BIBLIOGRAPHY AS TOPIC"[MESH] OR "INFORMATION SEEKING BEHAVIOR"[MESH] OR "DATABASE SEARCH"[TIAB] OR "DATABASE SEARCHES"[TIAB] OR "DATABASE SEARCHING"[TIAB] OR "LITERATURE SEARCH"[TIAB] OR "LITERATURE SEARCHES"[TIAB] OR "LITERATURE SEARCHING"[TIAB] OR "SEARCH RESULT"[TIAB] OR "SEARCH RESULTS"[TIAB] OR "SEARCH STRATEGY"[TIAB] OR "SEARCH STRATEGIES"[TIAB] OR "SEARCH STRING"[TIAB] OR "SEARCH STRINGS"[TIAB] OR "SEARCH PROCESS"[TIAB] OR "SEARCH PROCESSES"[TIAB] OR "INFORMATION SEARCH"[TIAB] OR "INFORMATION SEARCHES"[TIAB] OR "INFORMATION SEARCHING"[TIAB] OR "INFORMATION SEEKING"[TIAB] OR "INFORMATION FINDING"[TIAB] OR "LITERACY SKILLS"[TIAB]))	228,053
3	(("QUALITY IMPROVEMENT"[MESH] OR "EDUCATIONAL MEASUREMENT"[MESH] OR PROMOTION[TITLE] OR PROMOTE[TITLE] OR IMPROVEMENT[TITLE] OR IMPROVE[TITLE] OR INCREASE[TITLE] OR ENHANCEMENT[TITLE] OR ENHANCE[TITLE] OR DEVELOPMENT[TITLE] OR DEVELOP[TITLE] OR UPGRADE[TITLE] OR SUCCESS[TITLE] OR SUCCESSFUL[TITLE] OR SUCCESSFULLY[TITLE] OR ADVANCE[TITLE] OR ADVANCEMENT[TITLE] OR BENEFIT[TITLE] OR GAIN[TITLE] OR PROGRESS[TITLE] OR ACHIEVEMENT[TITLE] OR ACHIEVE[TITLE] OR BOOST[TITLE]))	994,330

Supplemental content to
J Med Libr Assoc. Oct;108(4):dx.doi.org/10.5195/jmla.2020.954
www.jmla.mlanet.org
© Hirt, Nordhausen, Meichlinger, Braun, Zeller, Meyer 2020

4	(("RANDOMIZED CONTROLLED TRIALS AS TOPIC"[MESH] OR "RANDOMIZED CONTROLLED TRIAL"[PT] "CONTROLLED CLINICAL TRIALS AS TOPIC"[MESH] OR "CONTROLLED CLINICAL TRIAL"[PT] OR "CROSS- OVER STUDIES"[MESH] OR TRIAL[TIAB] OR TRIALS[TIAB] OR STUDY[TIAB] OR STUDIES[TIAB] OR EXAMINATION[TIAB] OR EXAMINATIONS[TIAB] OR EXAMINATE[TIAB] OR EXPLORATORY[TIAB] OR INQUIRY[TIAB] OR INQUIRIES[TIAB] OR INVESTIGATION[TIAB] OR INVESTIGATIONS[TIAB] OR INVESTIGATE[TIAB] OR APPROACH[TIAB] OR DESIGN[TIAB] OR RANDOM*[TIAB] OR CLUSTER- RANDOM*[TIAB] OR "STEPPED WEDGE"[TIAB] OR STEPPED-WEDGE[TIAB] OR QUASI-EXPERIMENTAL[TIAB] OR EXPERIMENT[TIAB] OR EXPERIMENTS[TIAB] OR EXPERIMENTAL[TIAB] OR PRE- POST[TIAB] OR "PRE POST"[TIAB] OR CROSSOVER[TIAB]))	12,124,082
5	(("HEALTH PERSONNEL"[MESH] OR "HEALTH OCCUPATIONS"[MESH] OR STUDENTS[MESH] OR "HEALTH PERSONNEL"[TIAB] OR "HEALTH PROFESSIONAL"[TIAB] OR "HEALTH PROFESSIONALS"[TIAB] OR NURS*[TIAB] OR LIBRARI*[TIAB] OR STUDENT[TIAB] OR STUDENTS[TIAB] OR MIDWIFE*[TIAB] OR PSYCHOLOG*[TIAB] OR PSYCHOTHERAP*[TIAB] OR PHARMACY[TIAB] OR PHARMACEUTIC*[TIAB] OR "INFORMATION SPECIALIST"[TIAB] OR "INFORMATION SPECIALISTS"[TIAB] OR MEDICINE[TIAB] OR MEDICAL[TIAB] OR DOCTOR[TIAB] OR DOCTORS[TIAB] OR CLINIC[TIAB] OR CLINICS[TIAB] OR CLINICAL[TIAB] OR CLINICIAN[TIAB] OR CLINICIANS[TIAB] OR GRADUATE[TIAB] OR GRADUATES[TIAB] OR UNDERGRADUATE[TIAB] OR UNDERGRADUATES[TIAB] OR "PHYSICAL THERAPY"[TIAB] OR "PHYSICAL THERAPIES"[TIAB] OR PHYSIOTHERAP*[TIAB] OR THERAP*[TIAB] OR PARAMEDIC*[TIAB] OR PSYCHOLOGIST[TIAB] OR PSYCHOLOGISTS[TIAB] OR PSYCHOTHERAPIST[TIAB] OR PSYCHOTHERAPISTS[TIAB] OR "HEALTH SCIENCE"[TIAB] OR "HEALTH SCIENCES"[TIAB] OR "HEALTH SCIENTIST"[TIAB] OR "HEALTH SCIENTISTS"[TIAB] OR "HEALTH RESEARCHER"[TIAB] OR "HEALTH RESEARCHERS"[TIAB]))	8,164,854
6	#1 AND #2 AND #3 AND #4 AND #5	1,889

Supplemental content to
J Med Libr Assoc. Oct;108(4):dx.doi.org/10.5195/jmla.2020.954
www.jmla.mlanet.org
© Hirt, Nordhausen, Meichlinger, Braun, Zeller, Meyer 2020

PsycINFO via Ovid

Date: January 11, 2019

#	Enter	Hits
1	exp Education/ or exp Teaching Methods/ or exp Teaching/ or exp Curriculum/ or exp Mentor/ or exp Professional Development/ or exp Libraries/ or exp Information Services/ or exp Peers/ or "Peer review".ti. or "Peer reviews".ti. or "Peer reviewing".ti. or "Peer groups".ti. or Mentoring.ti. or Education.ti. or Educational.ti. or Educate.ti. or Educating.ti. or Teach.ti. or Teaching.ti. or Program.ti. or Programs.ti. or Programme.ti. or Programmes.ti. or Material.ti. or Materials.ti. or Flyer.ti. or Support.ti. or Supporting.ti. or Help.ti. or Helping.ti. or Manual.ti. or Manuals.ti. or Course.ti. or Courses.ti. or Training.ti. or Trainings.ti. or Train.ti. or Coaching.ti. or Coachings.ti. or Coach.ti. or Mentorship.ti. or Mentorships.ti. or Guide.ti. or Guides.ti. or Guidance.ti. or Guidances.ti. or Guideline.ti. or Guidelines.ti. or Instruction.ti. or Instructions.ti. or Instruct.ti. or Information.ti. or Inform.ti. or Consulting.ti. or Consultation.ti. or Consult.ti. or Schooling.ti. or Learning.ti. or Learn.ti. or Exercise.ti. or Tutor.ti. or Scholarship.ti.	749,462
2	exp Databases/ or exp Information Literacy/ or exp Information Seeking/ or "Database search".ti,ab. or "Database searches".ti,ab. or "Database searching".ti,ab. or "Literature search".ti,ab. or "Literature searches".ti,ab. or "Literature searching".ti,ab. or "Search result".ti,ab. or "Search results".ti,ab. or "Search strategy".ti,ab. or "Search strategies".ti,ab. or "Search string".ti,ab. or "Search strings".ti,ab. or "Search process".ti,ab. or "Search processes".ti,ab. or "Information search".ti,ab. or "Information searches".ti,ab. or "Information searching".ti,ab. or "Information seeking".ti,ab. or "Information finding".ti,ab. or "Literacy skills".ti,ab.	23,986
3	exp Educational Measurement/ or Promotion.ti. or Promote.ti. or Improvement.ti. or Improve.ti. or Increase.ti. or Enhancement.ti. or Enhance.ti. or Development.ti. or Develop.ti. or Upgrade.ti. or Success.ti. or Successful.ti. or Successfully.ti. or Advance.ti. or Advancement.ti. or Benefit.ti. or Gain.ti. or Progress.ti. or Achievement.ti. or Achieve.ti. or Boost.ti.	250,056
4	exp Experimental Methods/ or Trial.ti,ab. or Trials.ti,ab. or Study.ti,ab. or Studies.ti,ab. or Examination.ti,ab. or Examinations.ti,ab. or Examinate.ti,ab. or Exploratory.ti,ab. or Inquiry.ti,ab. or Inquiries.ti,ab. or Investigation.ti,ab. or Investigations.ti,ab. or Investigate.ti,ab. or Approach.ti,ab. or Design.ti,ab. or Random*.ti,ab. or Cluster-random*.ti,ab. or "Stepped wedge".ti,ab. or Stepped-wedge.ti,ab. or Quasi-experimental.ti,ab. or Experiment.ti,ab. or Experiments.ti,ab. or Experimental.ti,ab. or Pre-post.ti,ab. or "Pre post".ti,ab. or Crossover.ti,ab.	2,576,577
5	exp Health Personnel/ or exp Students/ or "Health Personnel".ti,ab. or "Health Professional".ti,ab. or "Health Professionals".ti,ab. or Nurs*.ti,ab. or Librari*.ti,ab. or Student.ti,ab. or Students.ti,ab. or Midwife*.ti,ab. or Psycholog*.ti,ab. or Psychotherap*.ti,ab. or Pharmacy.ti,ab. or Pharmaceutic*.ti,ab. or "Information specialist".ti,ab. or "Information specialists".ti,ab. or Medicine.ti,ab. or Medical.ti,ab. or Doctor.ti,ab. or Doctors.ti,ab. or Clinic.ti,ab. or Clinics.ti,ab. or Clinical.ti,ab. or Clinician.ti,ab. or Clinicians.ti,ab. or Graduate.ti,ab. or Graduates.ti,ab. or Undergraduate.ti,ab. or Undergraduates.ti,ab. or "Physical therapy".ti,ab. or "Physical therapies".ti,ab. or Physiotherap*.ti,ab. or Therap*.ti,ab. or Paramedic*.ti,ab. or Psychologist.ti,ab. or Psychologists.ti,ab. or	

Supplemental content to
J Med Libr Assoc. Oct;108(4):dx.doi.org/10.5195/jmla.2020.954
www.jmla.mlanet.org
© Hirt, Nordhausen, Meichlinger, Braun, Zeller, Meyer 2020

	Psychotherapist.ti,ab. or Psychotherapists.ti,ab. or "Health Science".ti,ab. or "Health Sciences".ti,ab. or "Health Scientist".ti,ab. or "Health Scientists".ti,ab. or "Health Researcher".ti,ab. or "Health Researchers".ti,ab.	
6	#1 AND #2 AND #3 AND #4 AND #5	400

Journal of the Medical Library Association

Supplemental content to
J Med Libr Assoc. Oct;108(4):dx.doi.org/10.5195/jmla.2020.954
www.jmla.mlanet.org
© Hirt, Nordhausen, Meichlinger, Braun, Zeller, Meyer 2020

Web of Science Core Collection

Date: January 11, 2019

#	Enter	Hits
1	TI=(Education OR "Teaching Materials" OR "Manuals as Topic" OR "Study Guide as Topic" OR Curriculum OR Mentoring OR "Library Services" OR "Peer Review" OR "Peer Group" OR "Peer review" OR "Peer reviews" OR "Peer reviewing" OR "Peer groups" OR Mentoring OR Education OR Educational OR Educate OR Educating OR Teach OR Teaching OR Program OR Programs OR Programme OR Programmes OR Material OR Materials OR Flyer OR Support OR Supporting OR Help OR Helping OR Manual OR Manuals OR Course OR Courses OR Training OR Trainings OR Train OR Coaching OR Coachings OR Coach OR Mentorship OR Mentorships OR Guide OR Guides OR Guidance OR Guidances OR Guideline OR Guidelines OR Instruction OR Instructions OR Instruct OR Information OR Inform OR Consulting OR Consultation OR Consult OR Schooling OR Learning OR Learn OR Exercise OR Tutor OR Scholarship)	3,313,389
2	TS=("Databases as Topic" OR "Information Literacy" OR "Search Engine" OR "Bibliography as Topic" OR "Information Seeking Behavior" OR "Database search" OR "Database searches" OR "Database searching" OR "Literature search" OR "Literature searches" OR "Literature searching" OR "Search result" OR "Search results" OR "Search strategy" OR "Search strategies" OR "Search string" OR "Search strings" OR "Search process" OR "Search processes" OR "Information search" OR "Information searches" OR "Information searching" OR "Information seeking" OR "Information finding" OR "Literacy skills")	105,080
3	TI=("Quality Improvement" OR "Educational Measurement" OR Promotion OR Promote OR Improvement OR Improve OR Increase OR Enhancement OR Enhance OR Development OR Develop OR Upgrade OR Success OR Successful OR Successfully OR Advance OR Advancement OR Benefit OR Gain OR Progress OR Achievement OR Achieve OR Boost)	3,700,770
4	TS=("Controlled Clinical Trials as Topic" OR "Controlled Clinical Trial" OR "Cross-Over Studies" OR Trial OR Trials OR Study OR Studies OR Examination OR Examinations OR Examinate OR Exploratory OR Inquiry OR Inquiries OR Investigation OR Investigations OR Investigate OR Approach OR Design OR Random* OR Cluster-random* OR "Stepped wedge" OR Stepped-wedge OR Quasi-experimental OR Experiment OR Experiments OR Experimental OR Pre-post OR "Pre post" OR Crossover)	25,534,079
5	TS=("Health Personnel" OR "Health Professional" OR "Health Professionals" OR Nurs* OR Librari* OR Student OR Students OR Midwife* OR Psycholog* OR Psychotherap* OR Pharmacy OR Pharmaceutic* OR "Information specialist" OR "Information specialists" OR Medicine OR Medical OR Doctor OR Doctors OR Clinic OR Clinics OR Clinical OR Clinician OR Clinicians OR Graduate OR Graduates OR Undergraduate OR Undergraduates OR "Physical therapy" OR "Physical therapies" OR Physiotherap* OR Therap* OR Paramedic* OR Psychologist OR Psychologists OR Psychotherapist OR Psychotherapists OR "Health Science" OR "Health Sciences" OR "Health Scientist" OR "Health Scientists" OR "Health Researcher" OR "Health Researchers")	7,361,620
6	#1 AND #2 AND #3 AND #4 AND #5	917

SC
Supplemental content to
J Med Libr Assoc. Oct;108(4):dx.doi.org/10.5195/jmla.2020.954
www.jmla.mlanet.org
© Hirt, Nordhausen, Meichlinger, Braun, Zeller, Meyer 2020

Embase via Ovid

Date: January 14, 2019

#	Enter	Hits
1	(exp Peer review/ OR exp Education/ OR exp Support group/ OR exp Guidance/ OR exp Guideline/ OR exp Learning/ OR "Peer review".ti OR "Peer reviews".ti OR "Peer reviewing".ti OR "Peer groups".ti OR Mentoring.ti OR Education.ti OR Educational.ti OR Educate.ti OR Educating.ti OR Teach.ti OR Teaching.ti OR Program.ti OR Programs.ti OR Programme.ti OR Programmes.ti OR Material.ti OR Materials.ti OR Flyer.ti OR Support.ti OR Supporting.ti OR Help.ti OR Helping.ti OR Manual.ti OR Manuals.ti OR Course.ti OR Courses.ti OR Training.ti OR Trainings.ti OR Train.ti OR Coaching.ti OR Coachings.ti OR Coach.ti OR Mentorship.ti OR Mentorships.ti OR Guide.ti OR Guides.ti OR Guidance.ti OR Guidances.ti OR Guideline.ti OR Guidelines.ti OR Instruction.ti OR Instructions.ti OR Instruct.ti OR Information.ti OR Inform.ti OR Consulting.ti OR Consultation.ti OR Consult.ti OR Schooling.ti OR Learning.ti OR Learn.ti OR Exercise.ti OR Tutor.ti OR Scholarship.ti)	2,464,950
2	(exp Database/ OR exp Search engine/ OR exp Information retrieval/ OR exp Information seeking/ OR exp Information literacy/ OR "Database search".ti,ab OR "Database searches".ti,ab OR "Database searching".ti,ab OR "Literature search".ti,ab OR "Literature searches".ti,ab OR "Literature searching".ti,ab OR "Search result".ti,ab OR "Search results".ti,ab OR "Search strategy".ti,ab OR "Search strategies".ti,ab OR "Search string".ti,ab OR "Search strings".ti,ab OR "Search process".ti,ab OR "Search processes".ti,ab OR "Information search".ti,ab OR "Information searches".ti,ab OR "Information searching".ti,ab OR "Information seeking".ti,ab OR "Information finding".ti,ab OR "Literacy skills".ti,ab)	472774
3	(Promotion.ti OR Promote.ti OR Improvement.ti OR Improve.ti OR Increase.ti OR Enhancement.ti OR Enhance.ti OR Development.ti OR Develop.ti OR Upgrade.ti OR Success.ti OR Successful.ti OR Successfully.ti OR Advance.ti OR Advancement.ti OR Benefit.ti OR Gain.ti OR Progress.ti OR Achievement.ti OR Achieve.ti OR Boost.ti)	1,001,928
4	(exp Action Research/ OR exp Controlled study/ OR exp Experimental study/ OR exp Quasi experimental study/ OR exp Control group/ OR exp Crossover procedure/ OR exp Single blind procedure/ OR exp Double blind procedure/ OR exp Triple blind procedure/ OR exp Experimental design/ OR exp Nonequivalent control group/ OR exp Parallel design/ OR Trial.ti,ab OR Trials.ti,ab OR Study.ti,ab OR Studies.ti,ab OR Examination.ti,ab OR Examinations.ti,ab OR Examinate.ti,ab OR Exploratory.ti,ab OR Inquiry.ti,ab OR Inquiries.ti,ab OR Investigation.ti,ab OR Investigations.ti,ab OR Investigate.ti,ab OR Approach.ti,ab OR Design.ti,ab OR Random*.ti,ab OR Cluster-random*.ti,ab OR "Stepped wedge".ti,ab OR Stepped- wedge.ti,ab OR Quasi-experimental.ti,ab OR Experiment.ti,ab OR Experiments.ti,ab OR Experimental.ti,ab OR Pre-post.ti,ab OR "Pre post".ti,ab OR Crossover.ti,ab)	16,702,951

Supplemental content to
J Med Libr Assoc. Oct;108(4):dx.doi.org/10.5195/jmla.2020.954
www.jmla.mlanet.org
© Hirt, Nordhausen, Meichlinger, Braun, Zeller, Meyer 2020

5	(exp Health care personnel/ OR exp Health student/ OR exp Research student/ OR exp Graduate student/ OR exp Undergraduate student/ OR exp PhD student/ OR exp Psychologist/ OR exp Librarian/ OR "Health Personnel".ti,ab OR "Health Professional".ti,ab OR "Health Professionals".ti,ab OR Nurs*.ti,ab OR Librari*.ti,ab OR Student.ti,ab OR Students.ti,ab OR Midwife*.ti,ab OR Psycholog*.ti,ab OR Psychotherap*.ti,ab OR Pharmacy.ti,ab OR Pharmaceutic*.ti,ab OR "Information specialist".ti,ab OR "Information specialists".ti,ab OR Medicine.ti,ab OR Medical.ti,ab OR Doctor.ti,ab OR Doctors.ti,ab OR Clinic.ti,ab OR Clinics.ti,ab OR Clinical.ti,ab OR Clinician.ti,ab OR Clinicians.ti,ab OR Graduate.ti,ab OR Graduates.ti,ab OR Undergraduate.ti,ab OR Undergraduates.ti,ab OR "Physical therapy".ti,ab OR "Physical therapies".ti,ab OR Physiotherap*.ti,ab OR Therap*.ti,ab OR Paramedic*.ti,ab OR Psychologist.ti,ab OR Psychologists.ti,ab OR Psychotherapist.ti,ab OR Psychotherapists.ti,ab OR "Health Science".ti,ab OR "Health Sciences".ti,ab OR "Health Scientist".ti,ab OR "Health Scientists".ti,ab OR "Health Researcher".ti,ab OR "Health Researchers".ti,ab)	9,726,521
6	#1 AND #2 AND #3 AND #4 AND #5	2,512
