# Supplementary material for: Educational interventions to improve literature searching skills in the health sciences: a scoping review
Source: J Med Libr Assoc. 2020 Oct 1;108(4):534–46. doi: 10.5195/jmla.2020.954 (PMC7524628; doi:10.5195/jmla.2020.954)
Supplement: Supplementary file 3 — Appendix C: Characteristics, interventions, outcomes, and main results of included studies [file jmla-108-4-534-s03.pdf]

JMLA Journal of the Medical Library Association

Supplemental content to
J Med Libr Assoc. Oct;108(4):dx.doi.org/10.5195/jmla.2020.954
www.jmla.mlanet.org
© Hirt, Nordhausen, Meichlinger, Braun, Zeller, Meyer 2020

Educational interventions to improve literature searching skills in the health sciences: a scoping review

Julian Hirt; Thomas Nordhausen; Jasmin Meichlinger; Volker Braun; Adelheid Zeller; Gabriele Meyer

APPENDIX C

Characteristics, interventions, outcomes, and main results of included studies

Reference	Country	Design	Setting	Participants	Number	Female/ male	Age (range, M)	Intervention group	Control group	Outcome measurement	Time of measurement	Main results
Sikora et al., 2019 [1]	Canada	Pre-post trial	University	Undergraduate or graduate health sciences or medical students	29	NI	NI	Scheduled individualized research consultations for students, performed by librarians	NA	Self-developed information literacy rubric for scoring of open- ended questions regarding the use of appropriate keywords and search strategies (0=insufficient, 1=acceptable, 2=superior), rated by the last author	Baseline (T0), post-intervention (T1)	<u>Use of appropriate keywords</u><br>T0: M=1, SD=0.66<br>T1: M=1.34, SD=0.72 (p>0.05)<br><u>Use of appropriate search strategies</u><br>T0: M=0.21, SD=0.41<br>T1: M=0.76, SD=0.79 (p=0.001)
Hobbs et al., 2015 [2]	USA	Pre-post trial	University	Senior undergraduate radiologic science students	17	NI	NI	Library instruction on planning literature searches, developing search strategies, searching health-related and medicine-related databases	NA	Questionnaire on knowledge and skills in information literacy developed by the health sciences librarian and the Radiologic Science Faculty (↑correctness), rating person(s) and rubric not described	Baseline (T0), post-intervention (T1)	<u>Database search skills</u><br>T0: Correct=29.4%<br>T1: Correct=94.1%<br><u>Developing search strategies</u><br>T0: Correct=82.3%<br>T1: Correct=100%<br><u>Search concepts</u><br>T0: Correct=94.1%<br>T1: Correct=100%<br><u>Database selection</u><br>T0: Correct=17.6%<br>T1: Correct=64.7%

JMLA Journal of the Medical Library Association

Supplemental content to
J Med Libr Assoc. Oct;108(4):dx.doi.org/10.5195/jmla.2020.954
www.jmla.mlanet.org
© Hirt, Nordhausen, Meichlinger, Braun, Zeller, Meyer 2020

Reference	Country	Design	Setting	Participants	Number	Female/ male	Age (range, M)	Intervention group	Control group	Outcome measurement	Time of measurement	Main results
Qureshi et al., 2015 [3]	Pakistan	Pre-post trial	University	Postgraduate dental students	42	20/22	NI	Workshop comprised 3 sessions of lectures and hands-on practice	NA	Questions of the Fresno Test tool (where to find evidence, 0–6, excellent=6; able to search PubMed, 0–8, excellent=8), rating person(s) not described	Baseline (T0), post-intervention (T1)	Where to find evidence<br>T0: M=1.2, SD=1.5<br>T1: M=2.0, SD=1.6 (*p*=0.002)<br>Able to search PubMed<br>T0: M=0.2, SD=0.8<br>T1: M=1.6, SD=2.4 (*p*=0.003)
Brettle and Raynor, 2013 [4]	UK	RCT	University	Undergraduate nursing students	55 (IG: 26; CG: 29)	NI	18–40, NI	Online in-house information literacy tutorial (session 1) and follow-up information skills session (face-to-face) after one month (session 2)	Face-to-face session, delivered by the nursing subject librarian (session 1) and follow-up information skills session	Test of skills to search for evidence via CINAHL concerning specific research questions using a rubric identifying key features in the search strategy (scoring of use of synonyms, any truncation, correct truncation, correct Boolean OR, correct Boolean AND [each 1 point], correct keywords [1 point for each up to 3], additional feature; e.g., limits, nesting [1 point for each up to 2], 0–10, ↑correctness), rated independently by 2 researchers, disagreements resolved through discussion	Baseline session 1 (T0), post-intervention session 1 (T1), Baseline session 2 (T2), post-intervention session 2 (T3)	IG<br>T0: M=0.35, SD=0.88<br>T1: M=1.77, SD=2.24<br>T0 vs. T1: *p*=0.001<br>T3: M=1.25, SD=1.48<br>T4: M=3.06, SD=1.7<br>CG<br>T1: M=2.23, SD=1.99<br>T0 vs. T1: *p*<0.001<br>T2: M=1.56, SD=1.54<br>T3: M=2.56, SD=1.86, SD=2.56<br>IG vs. CG<br>T0: *p*=0.588<br>T1: *p*=0.263

© Hirt, Nordhausen, Meichlinger, Braun, Zeller, Meyer 2020

Reference	Country	Design	Setting	Participants	Number	Female/ male	Age (range, M)	Intervention group	Control group	Outcome measurement	Time of measurement	Main results
Carlock and Anderson, 2007 [5]	USA	CT	University	Undergraduate nursing students	90 (IG: 60; KG: 30)	84/6	9–52, 27	Librarian instruction including homework and in-class assignment	No intervention	Self-created rubric comparing the search history of a predefined search in CINAHL against the rubric measuring 5 criteria (subject heading search, selection of subject headings, use of subheadings, combining of terms, use of limits, 0–100%, ↑correctness), rating person(s) not described	Baseline (T0), post-intervention (T1)	IG<br>T0: M=60.6% T1: M=88%<br>CG<br>T0: M=45% T1: M=47%
Grant and Brettle, 2006 [6]	UK	Pre-post trial	University	Postgraduate students in research in health and social care	13	NI	NI	Self-developed web-based MEDLINE tutorial by an information specialist and tutor	NA	Modified Rosenberg assessment tool comprised a skills checklist (e.g., Boolean operators, MeSH/indexing terms, application of limits, and whether a manageable and relevant number references were retrieved, 1–16, ↑correctness), rating person(s) not described	Baseline (T0), post-intervention (T1), post-intervention (T2, 10 weeks later)	T0: M=4.58, SD=1.5<br>T1: M=6.45, SD=1.46<br>T2: M=9.70, SD=3.53 (*p*=0.001)<br>T0 vs. T1: *p*=0.04<br>T1 vs. T2: *p*=0.008
Gruppen, et al., 2005 [7]	USA	CT	University	Fourth-year medical students	92 (IG: 34; CG: 58)	NI	NI	Instructional intervention on EBM-based techniques for searching MEDLINE for evidence related to a clinical problem	No intervention	Structured clinical scenario (described in a publication) and scoring on literature search quality and search errors developed by librarians at the University of Michigan, based on a template designed by librarians at the University of Rochester (quality: score	Baseline (T0), post-intervention (T1, one month later)	Quality score (overall)<br>IG<br>T0: M=59.8, SD=25.5<br>T1: M=72.5, SD=21.3 MD: 12.7, SD: 23.9<br>CG<br>T0: M=60, SD=21.2<br>T1: M=59.3, SD=16.9 MD: -0.7, SD=22.4

Supplemental content to
J Med Libr Assoc. Oct;108(4):dx.doi.org/10.5195/jmla.2020.954
www.jmla.mlanet.org
© Hirt, Nordhausen, Meichlinger, Braun, Zeller, Meyer 2020

Reference	Country	Design	Setting	Participants	Number	Female/ male	Age (range, M)	Intervention group	Control group	Outcome measurement	Time of measurement	Main results
								taught by medical librarians		max. 95, ↑quality; search errors: ↓correctness), rated by 2 librarians		IG vs. CG<br>T0, MD: -0.2 (95 % CI: -11.3 to 10.9, $p \geq 0.05$)<br>T1: MD: 13.2 (95 % CI: 4.1 to 22.3, $p<0.05$)<br>Search errors (total)<br>IG: M=4.4, SD=3.3 CG: M=6.2, SD=2.8<br>MD: -1.8, 95 % CI: -0.4 to -3.2, $p<0.05$
Rosenfeld et al., 2002 [8]	USA	Pre-post trial	Intensive care unit	Intensive care unit nurses	36	NI	20–>50, NI	Educational sessions complemented by a web-tutorial regarding information literacy competencies, performed by the medical librarian	NA	Self-defined, point-based competency rating scale (executing a search, proper use of subject headings, use of focusing [each 1 point], searching more than 1 database [3 points], using limits and Boolean operators [2 points], using keywords if no subject heading existed or in addition to subject headings [1 point], improper use of commands [minus 1 point], 0=no competency, 1–3=beginner, 4–6=intermediate, 7–9=advanced), each search rated by 2 medical librarians independently	Baseline (T0), post-intervention (T1)	T0<br>No competency: n=35<br>Intermediate: n=1<br>T1<br>No competency: n=21<br>Beginner: n=4<br>Intermediate: n=4<br>Advanced: n=3<br>Note: some of the data on searches were lost when the medical library server crashed.

SC
Supplemental content to
J Med Libr Assoc. Oct;108(4):dx.doi.org/10.5195/jmla.2020.954
www.jmla.mlanet.org
© Hirt, Nordhausen, Meichlinger, Braun, Zeller, Meyer 2020

Reference	Country	Design	Setting	Participants	Number	Female/ male	Age (range, M)	Intervention group	Control group	Outcome measurement	Time of measurement	Main results
Vogel et al., 2002 [9]	USA	Pre-post trial	Hospital	Second-year medicine residents	42	26/16	30	Workshop on using Ovid's version of MEDLINE	NA	Participants completed the MEDLINE performance checklist (documentation of searching, relevance of retrieved citations [each 2 items], and searching strategies [6 items], each item rated as correct [fulfilled at least once per search] or incorrect), rated by the first author (assistant professor of medicine)	Baseline (T0), post-intervention (T1, directly after workshop), and post-intervention (T2, 1 to 11 months after workshop)	Significantly higher percentage of residents correctly used MEDLINE searching skills (p<0.05)<br>Note: only p values available.
Wallace et al., 2000 [10]	Australia	CT	University	Undergraduate nursing, health, and behavioral sciences students	300 (IG: 100; CG: 200)	NI	NI	Curriculum-integrated information literacy program	No intervention	Objective test of library catalog skills regarding 5 domains (selecting suitable command, responding to command appropriately, selecting appropriate information source, locating journal article, using citation to locate article [1 point each domain], score 0–5, ↑skills), rating person(s) not described	Baseline (T0), post-intervention (T1)	<u>IG</u><br>T0, M: 1.85<br>T1, M: 3.41<br><u>CG</u><br>T0, M: 1.85<br>T1, M: 2.36<br>Number of students who performed better, worse, or equal (matched results between baseline and post-intervention):<br>Baseline>post-intervention: n=3<br>Baseline<post-intervention: n=51<br>Baseline=post-intervention: n=1

SC
Supplemental content to
J Med Libr Assoc. Oct;108(4):dx.doi.org/10.5195/jmla.2020.954
www.jmla.mlanet.org
© Hirt, Nordhausen, Meichlinger, Braun, Zeller, Meyer 2020

Reference	Country	Design	Setting	Participants	Number	Female/ male	Age (range, M)	Intervention group	Control group	Outcome measurement	Time of measurement	Main results
Erickson and Warner, 1998 [11]	USA	RCT	Hospital	Residents in obstetrics and gynecology	31 (IG1: 11; IG2: 12; CG:8)	15/16	NI	IG1<br>Hands-on tutorial session on the use of MEDLINE by health sciences librarian with hands-on instruction<br>No intervention	IG2<br>Tutorial on the use of MEDLINE at prescribed sessions performed by health sciences librarian	MEDLINE search recall and precision rates of 4 searches, rated by faculty members	Baseline (T0: searches 1+2), post-intervention (T1: searches 3+4)	Recall IG1<br>T0, M: 16%<br>T1, M: 16%<br>(no statistical significance)<br>IG2<br>T0, M: 21%<br>T1, M: 28%<br>(no statistical significance)<br>CG
Grant et al., 1996 [12]	USA	Pre-post trial	University	Pharmacy students	48	NI	NI	Lecture on systematic approach in combination with an online demonstration with Ovid to develop search strategies and homework assignments to perform a literature search	NA	Evaluation of 2 written search strategies (1 sensitive, 1 specific) concerning a predefined research question by pre-established scoring criteria (0–20, ↑correctness), rated by study authors (profession not described); different research question for each evaluation point	Baseline (T0), post-intervention (11 weeks later)	Creating sensitive search strategy<br>T0: 8.2, SD=2.2<br>T1: M=19.1, SD=1.6<br>Creating specific search strategy<br>T0: M=5.7, SD=3.3<br>T1: M=17.7, SD=3.3

JMLA Journal of the Medical Library Association

Supplemental content to
J Med Libr Assoc. Oct;108(4):dx.doi.org/10.5195/jmla.2020.954
www.jmla.mlanet.org
© Hirt, Nordhausen, Meichlinger, Braun, Zeller, Meyer 2020

Reference	Country	Design	Setting	Participants	Number	Female/ male	Age (range, M)	Intervention group	Control group	Outcome measurement	Time of measurement	Main results
Haynes et al., 1993 [13]	Canada	RCT	Hospital	Physicians and physicians-in-training	264 (IG: 130; CG: 134)	NI	NI	Feedback on the first 10 searches and assignment by a clinical MEDLINE preceptor	No intervention	Participants performed 10 MEDLINE searches concerning individual research questions; the percentage of successful searches was defined if at least 1 relevant reference was retrieved (score 5–7 on 1–7 relevance scale, ↑relevance), evaluated for 1st, 4th, and 8th search, relevance rated by a clinician	Baseline (T0, after the first search), post-intervention (T1, after 4 searches), post-intervention (T2, after 8 searches)	IG<br>T0: 65%<br>T1: 71%<br>T2: 80%<br>CG<br>T0: 65%<br>T1: 75%<br>T2: 72%
Bradigan and Mularski (1989) [14]	USA	Pre-post trial	University	Second-year medical students	9	NI	NI	Mini module courses performed by 2 librarian instructors	NA	Number of correct answers, 3 questions on the ability to extract important concepts in a statement of a medical problem to be searched online (5 questions on the use of Boolean/proximity operators, 1=correct answer, 0=incorrect answer, ↑correctness), rating person(s) not described	Baseline (T0), post-intervention (T1)	Ability to extract important concepts (total)<br>T0: M: .88<br>T1: M: 3<br>Use of Boolean and proximity operators (total)<br>T0: M=2.33<br>T1: M=4.77

Abbreviations: CT=Controlled trial; CG=Control group; CI=Confidence interval; EBM=Evidence-based medicine; IG=Intervention group; M=Mean; MD=Mean difference; MeSH=Medical Subject Headings; NA=Not applicable; NI=No information available; *p*=*p* value; OR=Odds ratio; RCT=Randomized controlled trial; SD=Standard deviation; SE=Standard error; T0=Baseline measurement; T1=Post-intervention measurement; vs=Versus.
Notes: ↑/↓=higher values indicate better outcomes; Underlined outcomes in case of multiple outcomes per study.

SC
Supplemental content to
J Med Libr Assoc. Oct;108(4):dx.doi.org/10.5195/jmla.2020.954
www.jmla.mlanet.org
© Hirt, Nordhausen, Meichlinger, Braun, Zeller, Meyer 2020

REFERENCES

1. Sikora L, Fournier K, Rebner J. Exploring the impact of individualized research consultations using pre and posttesting in an academic library: a mixed methods study. Evid Based Libr Inf Pract. 2019;14(1):2–21. DOI: http://dx.doi.org/10.18438/eblip29500.
2. Hobbs DL, Guo R, Mickelsen W, Wertz CI. Assessment of library instruction to develop student information literacy skills. Radiol Technol. 2015 Jan–Feb;86(3):344–9.
3. Qureshi A, Bokhari SAH, Pirvani M, Dawani N. Understanding and practice of evidence based search strategy among postgraduate dental students: a preliminary study. J Evid Based Dent Pract. 2015 Jun;15(2):44–9.
4. Brettle A, Raynor M. Developing information literacy skills in pre-registration nurses: an experimental study of teaching methods. Nurse Educ Today. 2013 Feb;33(2):103–9.
5. Carlock D, Anderson J. Teaching and assessing the database searching skills of student nurses. Nurs Educ. 2007 Nov–Dec;32(6):251–5.
6. Grant MJ, Brettle AJ. Developing and evaluating an interactive information skills tutorial. Health Inf Libr J. 2006 Jun;23(2):79–86.
7. Gruppen LD, Rana GK, Arndt TS. A controlled comparison study of the efficacy of training medical students in evidence-based medicine literature searching skills. Acad Med. 2005 Oct;80(10):940–4.
8. Rosenfeld P, Salazar-Riera N, Vieira D. Piloting an information literacy program for staff nurses: lessons learned. Comput Inform Nurs. 2002 Nov–Dec;20(6):236–41.
9. Vogel EW, Block KR, Wallingford KT. Finding the evidence: teaching medical residents to search MEDLINE. J Med Libr Assoc. 2002 Jul;90(3):327–30.
10. Wallace MC, Shorten A, Crookes PA. Teaching information literacy skills: an evaluation. Nurse Educ Today. 2000 Aug;20(6):485–9.
11. Erickson S, Warner ER. The impact of an individual tutorial session on MEDLINE use among obstetrics and gynaecology residents in an academic training programme: a randomized trial. Med Educ. 1998 May;32(3):269–73.
12. Grant KL, Herrier RN, Armstrong EP. Teaching a systematic search strategy improves literature retrieval skills of pharmacy students. Am J Pharm Educ. 1996;60(3):281–6.
13. Haynes RB, Johnston ME, McKibbon KA, Walker CJ, Willan AR. A program to enhance clinical use of MEDLINE. a randomized controlled trial. Online J Curr Clin Trials. 1993 May 11;doc no. 56.
14. Bradigan PS, Mularski CA. End-user searching in a medical school curriculum: an evaluated modular approach. Bull Med Libr Assoc. 1989 Oct;77(4):348–56.
