# Supplementary material for: Educational interventions to improve literature searching skills in the health sciences: a scoping review
Source: J Med Libr Assoc. 2020 Oct 1;108(4):534–46. doi: 10.5195/jmla.2020.954 (PMC7524628; doi:10.5195/jmla.2020.954)
Supplement: Supplementary file 4 — Appendix D: Detailed reporting assessment using the Template for Intervention Description and Replication (TIDieR) [file jmla-108-4-534-s04.pdf]

JMLA Journal of the Medical Library Association

Supplemental content to
J Med Libr Assoc. Oct;108(4):dx.doi.org/10.5195/jmla.2020.954
www.jmla.mlanet.org
© Hirt, Nordhausen, Meichlinger, Braun, Zeller, Meyer 2020

Educational interventions to improve literature searching skills in the health sciences: a scoping review

Julian Hirt; Thomas Nordhausen; Jasmin Meichlinger; Volker Braun; Adelheid Zeller; Gabriele Meyer

APPENDIX D

Detailed reporting assessment using the Template for Intervention Description and Replication (TIDieR)

By using the Template for Intervention Description and Replication (TIDieR) checklist, the authors intended to describe the reporting state concerning the interventions. Therefore, we defined a set of minimum required information that needed to be contained in the published research article to fulfil the criteria CReDECI 2 and TIDIieR.

TIDieR checklist	Y/N/NA	Quote and/or comment
Sikora et al., 2019 [1]		
What<br>Materials: Describe any physical or informational materials used in the intervention, including those provided to participants or used in intervention delivery or in training of intervention providers. Provide information on where the materials can be accessed (such as online appendix, uniform resource locator [URL])<br>Minimum required information: Material and content of the material used to conduct the intervention needed to be described	Y	Quote: Librarians were asked to use a Search Strategy Worksheet (supplemental Appendix A) with every student they met during an individualized research consultation (IRC) for the duration of the study, whether they were participating in the study or not. This worksheet is frequently used during regular IRCs at this library, outside the scope of this study, therefore, no training of the librarians was required.
What<br>Procedures: Describe each of the procedures, activities, and/or processes used in the intervention, including any enabling or support activities<br>Minimum required information: Procedure to conduct the intervention needed to be described	N	Comment: No information available.

SC
Supplemental content to
J Med Libr Assoc. Oct;108(4):dx.doi.org/10.5195/jmla.2020.954
www.jmla.mlanet.org
© Hirt, Nordhausen, Meichlinger, Braun, Zeller, Meyer 2020

TIDieR checklist	Y/N/NA	Quote and/or comment
Who provided<br>For each category of intervention provider (such as psychologist, nursing assistant), describe their expertise, background, and any specific training given<br>Minimum required information: Professional background and experience as well as specific training to conduct the intervention needed to be described	Y	Quote: Academic librarians…<br>For many librarians, IRCs are an integral part of their teaching repertoire…<br>This worksheet is frequently used during regular IRCs at this library, outside the scope of this study; therefore, no training of the librarians was required.
How<br>Describe the modes of delivery (such as face-to-face or by some other mechanism, such as Internet or telephone) of the intervention and whether it was provided individually or in a group<br>Minimum required information: Modes of delivery (facilitated/non-facilitated and individual/group) of the intervention needed to be described	Y	Quote: In-person, one-on-one tailored help is tremendously appreciated by students and should be kept as an additional service offered to students.
Where<br>Describe the type(s) of location(s) where the intervention occurred, including any necessary infrastructure or relevant features<br>Minimum required information: The institution needed to be described	N	Comment: No detailed information available.
When and how often<br>Describe the number of times the intervention was delivered and over what period of time including the number of sessions, their schedule, and their duration, intensity, or dose<br>Minimum required information: Frequency, duration, and length of the intervention needed to be described	N	Comment: There was probably one meeting with the students, but no detailed information available.

SC
Supplemental content to
J Med Libr Assoc. Oct;108(4):dx.doi.org/10.5195/jmla.2020.954
www.jmla.mlanet.org
© Hirt, Nordhausen, Meichlinger, Braun, Zeller, Meyer 2020

TIDieR checklist	Y/N/NA	Quote and/or comment
Tailoring<br>If the intervention was planned to be personalized, titrated, or adapted, then describe what, why, when, and how<br>Minimum required information: Measures to tailor the intervention needed to be described	Y	Quote: In-person, one-on-one tailored help is tremendously appreciated by students and should be kept as an additional service offered to students.
Modifications<br>If the intervention was modified during the course of the study, describe the changes (what, why, when, and how)<br>Minimum required information: Modifications of the intervention needed to be described	–	Comment: No information available.
How well<br>Planned: If intervention adherence or fidelity was assessed, describe how and by whom, and if any strategies were used to maintain or improve fidelity, describe them<br>Minimum required information: Measures to improve intervention fidelity needed to be described	–	Comment: No information available.
How well<br>Actual: If intervention adherence or fidelity was assessed, describe the extent to which the intervention was delivered as planned<br>Minimum required information: Intervention fidelity needed to be described	–	Comment: No information available.
Hobbs et al., 2015 [2]		
What	N	Comment: No information available.
What	Y	Quote: Students were given library instruction on planning literature searches, developing search strategies, searching health-related and medical-related databases to locate radiologic science literature, evaluating quality of information, and using EndNote, a software program that allows users to format references in different citation

Supplemental content to
J Med Libr Assoc. Oct;108(4):dx.doi.org/10.5195/jmla.2020.954
www.jmla.mlanet.org
© Hirt, Nordhausen, Meichlinger, Braun, Zeller, Meyer 2020

TIDieR checklist	Y/N/NA	Quote and/or comment
		styles (Thomson Reuters). Instruction was provided to demonstrate methodology in finding peer-reviewed articles in professional journals. During the library instruction, students completed hands-on exercises to practice what they learned.
Who provided	N	Quote: Health sciences librarians have provided the Idaho State University radiologic science students with library instruction.<br>Comment: No detailed information about the expertise of the health sciences librarian.
How	Y	Quote: This study consisted of a 120-minute hands-on library instruction and workshop session. Instruction was given to students once.
Where	Y	Quote: Idaho Health Sciences Library at Idaho State University in Pocatello, Idaho.
When and how often	Y	Quote: This study consisted of a 120-minute hands-on library instruction and workshop session. Instruction was given to students once.
Tailoring	–	Comment: No information available.
Modifications	–	Comment: No information available.
How well	–	Comment: No information available.
How well	–	Comment: No information available.
Qureshi et al., 2015 [3]		
What	N	Comment: No information available.
What	Y	Quote: The workshop comprised lectures and hands-on practice that included formulation of clinical question as well as strategies for searching and identifying the best possible clinical literature to answer the clinical question by identifying and applying possible types/categories of information sources and stating the advantages and disadvantages of each type of information source in terms of convenience. After an introduction about the role and applications of evidence-based practice (EBP), the participants were involved in a pre-workshop activity session. In this session, the participants were given an assessment tool that was used to measure participants' understanding and skills related to EBP. After pre-workshop (1st) activity session, in 2nd session participants were trained in and practiced completing a clinical case by creating and performing a search strategy to find appropriate evidence; and to describe methods to limit their search and explain reasons for it. A post-workshop (3rd) session was conducted to assess the changes in participants' knowledge and skills on the same real-life scenario that the participant chose in pre-workshop assessment phase.

SC
Supplemental content to
J Med Libr Assoc. Oct;108(4):dx.doi.org/10.5195/jmla.2020.954
www.jmla.mlanet.org
© Hirt, Nordhausen, Meichlinger, Braun, Zeller, Meyer 2020

TIDieR checklist	Y/N/NA	Quote and/or comment
Who provided	N	Comment: No information available.
How	Y	Quote: Pre- and post-workshop activity sessions were conducted, and assessment was made through a tool that was an adaptation of the Fresno Test.
Where	Y	Quote: University
When and how often	Y	Quote: Full-day workshop, of 3 sessions (2 hours each).
Tailoring	–	Comment: No information available.
Modifications	–	Comment: No information available.
How well	–	Comment: No information available.
How well	–	Comment: No information available.
Brettle and Raynor, 2013 [4]		
What	N	Quote: Students in the intervention group worked through the online tutorial, which they accessed via a designated area on the university's virtual learning environment (VLE)…<br>The tutorial was designed by a nursing subject librarian and learning technologist using Horizon Wimba and DreamWeaver software…<br>Library staff and a member of the research team (who had not developed the tutorial) facilitated by helping students with navigational or technical problems. To ensure further consistency each face-to-face session was provided by the same nursing subject librarian who had developed the tutorial.<br>Comment: No detailed information about the online tutorial and university's VLE.
What	Y	Quote: All students attended a library induction session at the beginning of the module, which covered basic library and information technology (IT) skills. Midway through the Foundations in Nursing Knowledge module, each group was scheduled to attend a 1-hour information literacy (IL) session (CG: face-to-face, IG: online tutorial) with a 1-hour follow-up session prior to the assessment hand-in date. These formed the basis of the study. To accommodate all the students, 4 sessions took place (1 intervention and 1 control per session) over 2 days. For the purpose of the study, students were allocated to the intervention group (online IL tutorial) or control (face-to-face teaching)…<br>A follow-up information skills session (face-to-face) was provided to both intervention and control groups 1 month after the first session (also 1 week before assignment hand-in date). At the end of this follow-up session, the control group was shown how to use

SC
Supplemental content to
J Med Libr Assoc. Oct;108(4):dx.doi.org/10.5195/jmla.2020.954
www.jmla.mlanet.org
© Hirt, Nordhausen, Meichlinger, Braun, Zeller, Meyer 2020

TIDieR checklist	Y/N/NA	Quote and/or comment
		the online tutorial. For the remainder of their course, all students had access to the tutorial…<br>Library staff and a member of the research team (who had not developed the tutorial) facilitated by helping students with navigational or technical problems with the online tutorial.
Who provided	N	Quote: Library staff and a member of the research team (who had not developed the tutorial). To ensure further consistency, each face-to-face session was provided by the same nursing subject librarian who had developed the tutorial.<br>Comment: No detailed information about the expertise of the involved person.
How	Y	Quote: Students in the control group received a face-to-face session, which replicated the content and structure of the tutorial…<br>The cohort was divided into 8 groups based on their nursing branches: 2×child, 2×mental health, 4×adult, with 11–12 students per group.
Where	N	Comment: No detailed information about the location of the sessions for control group (face-to-face).
When and how often	Y	Quote: Library induction session at the beginning of the module for all students. Midway through the Foundations in Nursing Knowledge module, each group was scheduled to attend a 1-hour IL session with a 1-hour follow up session. Afterward, 4 sessions took place (1 intervention and 1 control per session) over 2 days…<br>A follow-up information skills session (face-to-face) was provided to both intervention and control groups 1 month after the first session (also 1 week before assignment hand-in date). At the end of this follow-up session, the control groups were shown how to use the online tutorial.
Tailoring	–	Comment: No information available.
Modifications	–	Comment: No information available.
How well	–	Comment: No information available.
How well	Y	Comment: Discontinued intervention in IG: n=13 and in CG: n=8.
Carlock and Anderson, 2007 [5]		
What	N	Quote: No information available.
What	Y	Quote: The instruction was spread over 3 semesters and started in spring 2006: The librarian conducted lecture/hands-on instruction for the entire junior cohort of 90

Supplemental content to
J Med Libr Assoc. Oct;108(4):dx.doi.org/10.5195/jmla.2020.954
www.jmla.mlanet.org
© Hirt, Nordhausen, Meichlinger, Braun, Zeller, Meyer 2020

TIDieR checklist	Y/N/NA	Quote and/or comment
		students during their 1st professional development class. Students were introduced to CINAHL on the Ovid platform and were showed how to do a keyword search. The second session included a lecture/demonstration on how to conduct subject heading (controlled vocabulary) searches, followed by an hour of hands-on practice (in a group of 15 students); summer 2006 instruction: The next phase of instruction took place during summer 2006, when the librarian returned for an hour-long session in the psychiatric theory course. The librarian reviewed the major concepts taught previously, demonstrated subject searching procedures again, and pointed out the common mistakes students made in the performance assessment (in a group of 60 students); fall 2006 instruction: During group A's practical clinical time, the librarian conducted a 1.5-hour hands-on session in a computer-mediated classroom (in a group of 60 students).
Who provided	N	Quote: A librarian provided all instructions.<br>Comment: No detailed information about the expertise of the librarian.
How	Y	Quote: Lecture/hands-on instruction, carried out in groups of 90, 60, and 15 students.
Where	Y	Quote: At university…<br>During practical clinical time, the librarian conducted a session in a computer-mediated classroom…<br>The librarian returned for a session in the psychiatric theory course.
When and how often	Y	Quote: In total participants of IG received 4.5 hours of instruction with the librarian during the course of the fall, summer, and spring semesters; spring 2006 instruction: 2 sessions, each 30 minutes; summer 2006 instruction: 1-hour session; summer 2006 instruction: 1-hour lection for reviewing the major concepts taught previously; fall 2006 instruction: 1.5-hour hands on session in a computer-mediated classroom
Tailoring	–	Comment: No information available.
Modifications	–	Comment: No information available.
How well	–	Comment: No information available.
How well	–	Comment: No information available.
Grant and Brettle, 2006 [6]		
What	Y	Quote: The first session began with a lecture introducing students (n=21) to information systems (IS) theory (formulating a search question, selecting search terms, building up a search strategy, limiting searches) including an online demonstration. This was complemented with small group discussion and feedback of alternative information

SC
Supplemental content to
J Med Libr Assoc. Oct;108(4):dx.doi.org/10.5195/jmla.2020.954
www.jmla.mlanet.org
© Hirt, Nordhausen, Meichlinger, Braun, Zeller, Meyer 2020

TIDieR checklist	Y/N/NA	Quote and/or comment
		sources, and guided hands-on practice using the tutorial (http://www.fhsc.salford.ac.uk/hcprdu/litsearching.htm)…<br>The second session, attended by 13 students, sought to consolidate learning through small group work to address areas of confusion or ambiguity. Students were also given the opportunity to complete or revisit the tutorial, to repeat the exercises on alternative databases, possibly using a different search interface (e.g., SilverPlatter, WebSpirs, Dialog), and/or undertake their own searches in connection with the module assignment.
What	Y	Quote: The first session began with a lecture introducing students (n=21) to IS theory (formulating a search question, selecting search terms, building up a search strategy, limiting searches) including an online demonstration. This was complemented with small group discussion and feedback of alternative information sources, and guided hands-on practice using the tutorial (http://www.fhsc.salford.ac.uk/hcprdu/litsearching.htm)…<br>The 2nd session, attended by 13 students, sought to consolidate learning through small group work to address areas of confusion or ambiguity. Students were also given the opportunity to complete or revisit the tutorial, to repeat the exercises on alternative databases, possibly using a different search interface (e.g., SilverPlatter, WebSpirs, Dialog), and/or undertake their own searches in connection with the module assignment…<br>Students could access the tutorial at any time between the first session and the completion of their end-of-module assignment. Additionally, they could request feedback on their searches and obtain general advice on the development of searches prepared as part of their course assessment.
Who provided	N	Quote: Each session was facilitated by an information specialist and support tutor.<br>Comment: No detailed information about the expertise of the information specialist.
How	Y	Comment: Face-to-face, in groups.
Where	Y	Quote: University.<br>Comment: The first lecture included an online demonstration, for which computers must have been available as infrastructure.
When and how often	Y	Quote: 12-week module, with 2 session in week 2 and 3 were allocated, each session lasted approximately 3 hours.

Supplemental content to
J Med Libr Assoc. Oct;108(4):dx.doi.org/10.5195/jmla.2020.954
www.jmla.mlanet.org
© Hirt, Nordhausen, Meichlinger, Braun, Zeller, Meyer 2020

TIDieR checklist	Y/N/NA	Quote and/or comment
Tailoring	Y	Quote: Additionally, they could request feedback on their searches and obtain general advice on the development of searches prepared as part of their course assessment.
Modifications	–	Comment: No information available.
How well	–	Comment: No information available.
How well	–	Comment: No information available.
Gruppen et al., 2005 [7]		
What	N	Quote: Appraisal guidelines from the *Journal of the American Medical Association*'s "Users' Guides to the Medical Literature" series provided the framework for judging the quality of the findings…<br>Computerized access to MEDLINE….<br>Students were presented with brief clinical scenarios.<br>Comment: No detailed information about informational materials available.
What	Y	Quote: The elective taught the principles of appraising the validity and applicability of medical research literature related to a variety of clinical questions, including those related to therapy, diagnostic tests, economic analysis, decision analysis, overviews, guidelines, clinical measurement, prognosis, quality of care, and causation/harm…<br>As part of the elective, each student was required to generate 5 clinical questions, based on their own interests and clinical experiences, and to investigate these questions using the evidence-based medicine (EBM) techniques taught in the elective. Each question was to reflect a different type of research literature (e.g., economic analysis, therapy, prognosis). The students searched the literature for relevant evidence, selected the article they considered most relevant, and appraised its validity and utility according to the guidelines provided in the elective. They finished by writing a brief summary of their analysis of each question…<br>One optional component of the elective for each of the 3 years was for students to train in the use of MEDLINE in an EBM context. The stated objectives for students in this session were to review the concepts and strategies of EBM, to formulate a clinical question and effective search strategy, to locate EBM literature in MEDLINE, and to briefly be introduced to other tools for EBM. The format of the elective consisted of brief lectures interspersed with guided hands-on practice taught by medical librarians (Arndt and Rana). Each student had computerized access to MEDLINE during the elective. Students were presented with brief clinical scenarios and applied the "Patient,

SC
Supplemental content to
J Med Libr Assoc. Oct;108(4):dx.doi.org/10.5195/jmla.2020.954
www.jmla.mlanet.org
© Hirt, Nordhausen, Meichlinger, Braun, Zeller, Meyer 2020

TIDieR checklist	Y/N/NA	Quote and/or comment
		Intervention, Comparison, Outcome" technique to formulate a clinical question. They then practiced translating the question into a MEDLINE search strategy using the Ovid search interface for MEDLINE. The instructors presented techniques for filtering retrieval of high-quality evidence during the practice sessions, which were also detailed in an accompanying handout.
Who provided	N	Quote: Training in the use of MEDLINE: Medical librarians.<br>Comment: No detailed information about the intervention provider of the other components of the elective and their expertise and the expertise of the medical librarians.
How	Y	Comment: Face-to-face, in group.
Where	Y	Quote: University…<br>Computerized access to MEDLINE.
When and how often	N	Quote: Four-week elective course in EBM: consisting of a 90-minute session on each literature type…<br>Training in the use of MEDLINE: consisting of a single 2-hour session at the beginning of the elective, before the principles of EBM were taught in-depth.<br>Comment: No detailed information about the number of literature types and associated sessions.
Tailoring	Y	Quote: Students self-selected to the optional MEDLINE search training session for each of the 3 years.
Modifications	–	Comment: No information available.
How well	–	Comment: No information available.
How well	–	Comment: No information available.
Rosenfeld et al., 2002 [8]		
What	Y	Quote: Before beginning the program, participants were given appropriate log-ons and instructions on remote access to library resources…<br>Participants were oriented to a range of electronic sources of information, both fee-based and free…<br>Initially, participants were given a set of sample clinical questions for the searches…<br>After initial training, participants were given "Clinical Question Forms" that included reminders and hints that reinforced what they had learned in the educational sessions. The form included a checklist of steps to perform when searching databases, such as

Supplemental content to
J Med Libr Assoc. Oct;108(4):dx.doi.org/10.5195/jmla.2020.954
www.jmla.mlanet.org
© Hirt, Nordhausen, Meichlinger, Braun, Zeller, Meyer 2020

TIDieR checklist	Y/N/NA	Quote and/or comment
		CINAHL and MEDLINE, as well as a listing of the most frequently used websites, such as Joint Accreditation Commission of Healthcare Organizations (JACHO), the National Institutes of Health (NIH), and specialty nursing organizations.
What	Y	Quote: Before the educational sessions, each nurse received technical instruction about logging onto the library website, obtaining proper authentication for off-site Internet connections, and using library resources…<br>Sessions focused on the need to develop a clinical question appropriate for searching, identifying appropriate sources (e.g., databases, websites) to obtain the information, conducting the search (using different search strategies), and obtaining the needed information. Nurses learned several strategies, including using correct subject headings and subheadings, exploding and focusing (as appropriate), searching keywords, combining Boolean operators, using appropriate limits, and understanding the value of searching more than one database…<br>In addition, participants learned how to obtain and assess the reliability of information through the World Wide Web.<br>Because access and availability do not always translate into conscientious or proper use of these technologies, participants were also prepared to critically appraise the information they obtained through computer technologies…<br>With the support and cooperation of nurse managers, participants had some free time to perform searches...<br>After initial training, participants were given "Clinical Question Forms" that included reminders and hints that reinforced what they had learned in the educational sessions. The form included a checklist of steps to perform when searching databases, such as CINAHL and MEDLINE, as well as a listing of the most frequently used websites, such as JACHO, NIH, and specialty nursing organizations. In addition, a web-based tutorial (http://library.med.nyu.edu/tutorials/nursing) was developed to reinforce understanding of search techniques and strategies and to facilitate at-home or library literature searches…<br>After the sessions, the medical librarian made weekly visits to the unit to monitor progress, answer questions, and promote the project.
Who provided	N	Quote: The medical librarian, with the assistance of the critical care instructor, provided scheduled unit-based training sessions using lecture/demonstration methods.<br>Comment: No detailed information about the providers of the intervention.

Supplemental content to
J Med Libr Assoc. Oct;108(4):dx.doi.org/10.5195/jmla.2020.954
www.jmla.mlanet.org
© Hirt, Nordhausen, Meichlinger, Braun, Zeller, Meyer 2020

TIDieR checklist	Y/N/NA	Quote and/or comment
How	Y	Quote: Five to 6 registered nurses (RNs) attended each initial training session… The staff development instructor and medical librarian conducted weekly sessions on the unit for 6 weeks, until all participants received hands-on training.
Where	Y	Quote: The decision was made to conduct the educational instruction on the patient care units rather than require staff to come to the library training classrooms. Based on experiences with staff nurses, the medical librarians believed that it would be more difficult to bring the RNs to classroom settings in the library and that unit-based instruction would provide a more effective use of the practitioner's time… A dedicated computer connected to the Internet and printer were available to participants in the unit.
When and how often	Y	Quote: Weekly sessions on the unit for 6 weeks, until all participants received hands-on training. The weekly sessions lasted approximately 1 hour.
Tailoring	–	Comment: No information available.
Modifications	–	Comment: No information available.
How well	–	Comment: No information available.
How well	–	Comment: No information available.
Vogel et al., 2002 [9]		
What	Y	Quote: In advance of the workshop, the residents were assigned to read a series of articles about using MEDLINE and searching to find answers to clinical questions… A handout contained materials from the didactic session, the clinical scenarios, and step-by-step instructions for the MEDLINE searches.
What	Y	Quote: Workshops started with a didactic presentation on MEDLINE searching. Topics included Medical Subject Headings (MeSH) versus text-word searching, "explode" feature, search term truncation, Boolean operators, and use of the methodological filters developed by Haynes et al. Lastly, we provided an overall approach for converting clinical questions from patient encounters into MEDLINE search strategies… The class then moved to the library computer center, where the residents were provided with personal computers for the remainder of the workshop. Searches were performed with Ovid's MEDLINE search interface. We guided the residents step by step in performing 2 MEDLINE searches for clinical questions from 2 hypothetical clinical scenarios, using the search functions and strategies described in the earlier didactic session…

SC
Supplemental content to
J Med Libr Assoc. Oct;108(4):dx.doi.org/10.5195/jmla.2020.954
www.jmla.mlanet.org
© Hirt, Nordhausen, Meichlinger, Braun, Zeller, Meyer 2020

TIDieR checklist	Y/N/NA	Quote and/or comment
		Finally, class participants independently performed practice searches from a 3rd scenario, with instructors available for assistance.
Who provided	N	Quote: Two instructors supported the workshop; one primarily led the step-by-step instructions, and the other provided individual assistance for participants as needed.<br>Comment: No detailed information about the background and the expertise of the instructors.
How	Y	Quote: In groups, with an average of 7 residents per workshop.
Where	Y	Quote: Library computer center, where the residents were provided with personal computers.
When and how often	Y	Quote: A 3-hour workshop, 6 times (once per month over a 6-month period)…<br>The workshop began with a 30-minute didactic presentation on MEDLINE searching.
Tailoring	–	Comment: No information available.
Modifications	–	Comment: No information available.
How well	–	Comment: No information available.
How well	–	Comment: No information available.
Wallace et al., 2000 [10]		
What	N	Quote: Within tutorials, students are provided with short articles from mainstream magazines and are asked to describe the article in general terms then list as many positive features (strengths) and as many negatives features (weaknesses) as they can (Learning Activity 3).<br>Comment: No further information about informational materials.
What	Y	Quote: There are 3 main assessment tasks each supported by a combination of learning activities. Learning Activity 1: Faculty librarian provides and lectures on how to read and understand the components of a bibliographic citation in order to find a library item. Students have scheduled time in the library. Learning Activity 2: Lecture about search strategies. The faculty librarian demonstrates the development and implementation of a search strategy related to a specific assignment topics set for this particular subject. This lecture can involve reinforcement of certain aspects of the use of the library catalog, use of databases and indexes. The tutors allocate tutorial time to the development of a search strategy, and there is structured time available in the library. Learning Activity 3: Occurred within the other activities, students are asked to describe short articles and are

JMLA Journal of the Medical Library Association

SC
Supplemental content to
J Med Libr Assoc. Oct;108(4):dx.doi.org/10.5195/jmla.2020.954
www.jmla.mlanet.org
© Hirt, Nordhausen, Meichlinger, Braun, Zeller, Meyer 2020

TIDieR checklist	Y/N/NA	Quote and/or comment
		then assisted to develop their "point-form" notes into sentences and paragraphs. As a result of this exercise students are able to write 1 general summary statement of their observations about the article and support this summary statement with reference to the strengths and weaknesses they have identified. (see http://dx.doi.org/10.1054/nedt.1999.0621)
Who provided	N	Quote: A professional faculty librarian provided lectures and demonstrations, a tutor allocated tutorial time.<br>Comment: No detailed information about the expertise of both.
How	Y	Quote: Face-to-face and in groups, as students have within the lectures time in the library, and the program is integrated into the curriculum.
Where	Y	Quote: All the learning activities were located in the library.
When and how often	N	Quote: 3 learning activities, Learning Activity 1 in week 2 of the autumn session, Learning Activity 2 in week 5 of autumn session, Learning Activity 3 occurred during the other activities.<br>Comment: No information about the length of the single lectures.
Tailoring	–	Comment: No information available.
Modifications	–	Comment: No information available.
How well	–	Comment: No information available.
How well	–	Comment: No information available.
Erickson and Warner, 1998 [11]		
What	Y	Quote: Residents received instructions for accessing the hospital's MEDLINE search engine using a log-on code developed for this study. Instructions were also posted at the MEDLINE terminals on the obstetric wards and at the library's reference desk.
What	Y	Quote: Individual tutorial with exposure to the following search techniques regarding the use of MEDLINE: (1) MeSH, (2) exploding, (3) restricting to focus, (4) subheadings, (5) text-word searching, (6) author searching, (7) limiting, (8) combining, (10) viewing, (11) printing, and (12) using backfiles…<br>The 11 residents in Group B received hands-on instruction, with the resident performing the searches. The 12 residents in Group C attended prescribed sessions with all searching conducted by the instructor.

Supplemental content to
J Med Libr Assoc. Oct;108(4):dx.doi.org/10.5195/jmla.2020.954
www.jmla.mlanet.org
© Hirt, Nordhausen, Meichlinger, Braun, Zeller, Meyer 2020

TIDieR checklist	Y/N/NA	Quote and/or comment
Who provided	Y	Quote: Health sciences librarian.<br>Comment: No detailed information about the expertise.
How	Y	Quote: Individually, face-to-face.
Where	N	Comment: No information about the location of the tutorial.
When and how often	Y	Quote: 1-hour tutorial
Tailoring	–	Comment: No information available.
Modifications	–	Comment: No information available.
How well	–	Comment: No information available.
How well	–	Comment: No information available.
Grant et al., 1996 [12]		
What	Y	Quote: Students received a homework assignment. Each homework assignment had 4 questions in which the students were to perform a literature search. They were required to print out their search strategies and list of articles the strategy retrieved…<br>This was combined with a 50-minute online demonstration with Ovid using a notebook computer connected to a local area network (LAN) line, an overhead projector, and LCD screen.
What	Y	Quote: 50-minute lecture on a systematic approach to developing search strategies was presented to the students. This was combined with a 50-minute online demonstration with Ovid. The primary goal of the lecture and demonstration was to emphasize the importance of a systematic approach to literature retrieval compared to haphazardly entering terms and scanning hundreds of articles. Secondary goals were to make sure the students understood the difference between using MEDLINE's controlled vocabulary and free-text searching; the inherent problems with MeSH terms; when to use MESH terms, free-text terms, subheadings, and explosions; and the appropriate use of Boolean operators. The students were shown that combining MeSH terms and free text is imperative to retrieve the maximum number of pertinent citations. The students were then shown appropriate ways to further refine the retrieval using limits such as "human," "English," and age groups. At the end of the demonstration, students received a homework assignment to practice the principles presented. Eight different homework assignment sets were distributed to the 48 students. The homework was designed to give examples of exhaustive and precise literature searches. Two weeks later, after the

Supplemental content to
J Med Libr Assoc. Oct;108(4):dx.doi.org/10.5195/jmla.2020.954
www.jmla.mlanet.org
© Hirt, Nordhausen, Meichlinger, Braun, Zeller, Meyer 2020

TIDieR checklist	Y/N/NA	Quote and/or comment
		homework was returned and graded, a 3rd 50-minute session reviewed the homework assignments.
Who provided	N	Comment: No information available.
How	Y	Quote: In groups, face-to-face
Where	N	Comment: No detailed information about the location where the sessions occurred.
When and how often	Y	Quote: Three 50-minute sessions: (1) lecture on a systematic approach to developing search strategies combined with (2) online demonstration with OVID, (3) 2 weeks later, review of the homework assignments.
Tailoring	–	Comment: No information available.
Modifications	–	Comment: No information available.
How well	–	Comment: No information available.
How well	–	Comment: No information available.
Haynes et al., 1993 [13]		
What	Y	Quote: Hardware for the study includes Zenith Model 159 XT-compatible microcomputers with 20 megabyte hard disks, Tandy 1200 baud modems, and HP QuietJet printers set up in each of the clinical areas. Software includes GRATEFUL MED 2.0 for searching the MEDLINE files, a customized Mirror (Cross-Talk clone communications software) script to retrieve specified full-text articles from BRS Colleague, and a literature critical appraisal program, LITEVAL. The software is accessed only through a purpose-built front-end program designed to collect data on who signs in, what question they want answered, if the question is about a specific patient, and, if so, the patient's name and hospital identification number. The front-end program records the time for all steps in the searching process and stores the search formulation and any citations and/or full-text articles retrieved. (However, we do not know how much time was spent on search preparation before signing on to the front-end program.) The user receives an immediate printout of any material that he or she wishes but the complete searches are stored on disk and collected daily for analysis. (see https://www.ncbi.nlm.nih.gov/pmc/articles/PMC2245175/)
What	N	Quote: On each of their 1st 10 searches, they received individualized feedback from a clinical preceptor experienced in MEDLINE searching. This feedback was completed and mailed each working day morning by a study librarian.<br>Comment: No detailed information about the content of the session.

SC
Supplemental content to
J Med Libr Assoc. Oct;108(4):dx.doi.org/10.5195/jmla.2020.954
www.jmla.mlanet.org
© Hirt, Nordhausen, Meichlinger, Braun, Zeller, Meyer 2020

TIDieR checklist	Y/N/NA	Quote and/or comment
Who provided	Y	Quote: Two study librarians (one of these audited each of the 1st 10 study group searches and sent a form to the searcher with comments and suggestions for improvement) and clinical MEDLINE preceptor (clinicians with proven searching skills (recall on test searches statistically indistinguishable from a research librarian) who had agreed to be "search coach" for several individuals in return for free MEDLINE searching from the study computers. Preceptors were assigned to participants in the same clinical discipline whenever possible.
How	Y	Quote: Feedback for the 1st 10 searches face-to-face individually.
Where	Y	Quote: Health sciences library of a tertiary-care teaching hospital with a medical school.
When and how often	Y	Quote: Feedback for the 1st 10 searches face-to-face individually.
Tailoring	–	Comment: No information available.
Modifications	–	Comment: No information available.
How well	–	Comment: No information available.
How well	–	Comment: No information available.
Bradigan and Mularski, 1989 [14]		
What	Y	Quote: Once the course description and outline were finalized, the librarians shared the materials with appropriate personnel in the College of Medicine, who used the description to advertise the mini module and handled the registration of students…<br>A demonstration of MEDLINE on Compact Cambridge's CD-ROM were held…<br>The instructors introduced BRS Colleague via a videotape and an online demonstration.
What	Y	Quote: A low instructor-to-student ratio was particularly important in order to give individualized attention during hands-on sessions…<br>In the first mini module class, the instructors conducted the administration of the pretest and provided lectures on background information and terminology, the basics of search strategy development (i.e., concept extraction and Boolean logic), and a demonstration of MEDLINE on Compact Cambridge's CD-ROM. The instructors developed 5 search problems on various topics, which they introduced to the students as the first assignment. Students were required to develop a keyword search strategy on one of the topics; they later executed it on their own time on the Compact Cambridge CD-ROM system located in the library's reference area. All health sciences librarians were aware of the assignments and assisted students at the reference desk when necessary. In the second class, students discussed their success and problems with the CD-ROM

SC
Supplemental content to
J Med Libr Assoc. Oct;108(4):dx.doi.org/10.5195/jmla.2020.954
www.jmla.mlanet.org
© Hirt, Nordhausen, Meichlinger, Braun, Zeller, Meyer 2020

TIDieR checklist	Y/N/NA	Quote and/or comment
		assignment. Students developed their own search strategies with BRS Colleague protocols, using 5 new search problems devised by the instructors and executed them in the microcomputer laboratory. Two students worked together on each personal computer during all online sessions; the instructors were available to provide assistance, as needed. Handouts on MeSH were distributed at the end of the second session. The instructors requested that the students read this material before the 3rd class. In the 3rd session, the instructors reviewed the concept of subject headings. Keyword searching and descriptor searching were explained in detail, and online examples were used to illustrate the differences in retrieval. The students developed strategies for new search problem assignments, using the MeSH terms and subheadings, and executed them online. Between the 3rd and the 4th classes, the students were to devise a tentative search strategy on a topic of personal interest to them. The instructors also distributed 20 search statements to students who did not choose their own topic. In the 4th session, the instructors reviewed the information from the 1st 3 sessions, and the class discussed the value of online searching in medical practice or research. Following completion of the post-test and evaluations, students developed final search strategies and executed them in the microcomputer laboratory.
Who provided	N	Quote: 2 librarian-instructors for the lectures…<br>In addition to the new part-time librarian, staff from all areas of the library assisted the instructors by staffing the Information Services Desk during the actual class period.<br>Comment: No detailed information about the expertise of the instructors.
How	Y	Quote: Prior to the 1st class, the librarians received a roster of 10 medical students, the class maximum based on course content, classroom size, and equipment availability.
Where	Y	Quote: Mini module lectures and search strategy planning were held in the library's classroom. Online time for student practice took place in the microcomputer laboratory on another floor. This division of learning experiences was necessary because the classroom was better organized and equipped for lecturing.
When and how often	Y	Quote: The short courses were offered on 4 consecutive Wednesday afternoons. Each session ran approximately 3 hours.
Tailoring	–	Comment: No information available.
Modifications	–	Comment: No information available.
How well	–	Comment: No information available.
How well	–	Comment: No information available.

JMLA Journal of the Medical Library Association

Supplemental content to
J Med Libr Assoc. Oct;108(4):dx.doi.org/10.5195/jmla.2020.954
www.jmla.mlanet.org
© Hirt, Nordhausen, Meichlinger, Braun, Zeller, Meyer 2020

Abbreviations: Y=Yes, reported; NA=Not applicable because the intervention was not tailored or modified; N=No, not reported; —=Unclear whether it was conducted.

REFERENCES

1. Sikora L, Fournier K, Rebner J. Exploring the impact of individualized research consultations using pre and posttesting in an academic library: a mixed methods study. Evid Based Libr Inf Pract. 2019;14(1):2—21. DOI: http://dx.doi.org/10.18438/eblip29500.
2. Hobbs DL, Guo R, Mickelsen W, Wertz CI. Assessment of library instruction to develop student information literacy skills. Radiol Technol. 2015 Jan—Feb;86(3):344—9.
3. Qureshi A, Bokhari SAH, Pirvani M, Dawani N. Understanding and practice of evidence based search strategy among postgraduate dental students: a preliminary study. J Evid Based Dent Pract. 2015 Jun;15(2):44—9.
4. Brettle A, Raynor M. Developing information literacy skills in pre-registration nurses: an experimental study of teaching methods. Nurse Educ Today. 2013 Feb;33(2):103—9.
5. Carlock D, Anderson J. Teaching and assessing the database searching skills of student nurses. Nurs Educ. 2007 Nov—Dec;32(6):251—5.
6. Grant MJ, Brettle AJ. Developing and evaluating an interactive information skills tutorial. Health Inf Libr J. 2006 Jun;23(2):79—86.
7. Gruppen LD, Rana GK, Arndt TS. A controlled comparison study of the efficacy of training medical students in evidence-based medicine literature searching skills. Acad Med. 2005 Oct;80(10):940—4.
8. Rosenfeld P, Salazar-Riera N, Vieira D. Piloting an information literacy program for staff nurses: lessons learned. Comput Inform Nurs. 2002 Nov—Dec;20(6):236—41.
9. Vogel EW, Block KR, Wallingford KT. Finding the evidence: teaching medical residents to search MEDLINE. J Med Libr Assoc. 2002 Jul;90(3):327—30.
10. Wallace MC, Shorten A, Crookes PA. Teaching information literacy skills: an evaluation. Nurse Educ Today. 2000 Aug;20(6):485—9.
11. Erickson S, Warner ER. The impact of an individual tutorial session on MEDLINE use among obstetrics and gynaecology residents in an academic training programme: a randomized trial. Med Educ. 1998 May;32(3):269—73.
12. Grant KL, Herrier RN, Armstrong EP. Teaching a systematic search strategy improves literature retrieval skills of pharmacy students. Am J Pharm Educ. 1996;60(3):281—6.
13. Haynes RB, Johnston ME, McKibbon KA, Walker CJ, Willan AR. A program to enhance clinical use of MEDLINE. a randomized controlled trial. Online J Curr Clin Trials. 1993 May 11;doc no. 56.
14. Bradigan PS, Mularski CA. End-user searching in a medical school curriculum: an evaluated modular approach. Bull Med Libr Assoc. 1989 Oct;77(4):348—56.
